# Development and clinical validation of a CRISPR/Cas9-engineered reporter phage cocktail for rapid detection of *Escherichia coli* in urine

**DOI:** 10.1128/spectrum.00996-26

**Published:** 2026-08-06

**Authors:** Zhiyun Hao, Qiang Zhao, Yi Zhong, Minwei Li, Chengbin Wang, Chi Wang

**Affiliations:** 1 PLA Medical School 104607 , Beijing, China; 2 Department of Clinical Laboratory Medicine, The First Medical Center of PLA General Hospital 651943, Beijing, China; City of Hope Department of Pathology, Duarte, California, USA

**Keywords:** reporter phage, CRISPR/Cas9, *Escherichia coli*, phage cocktail, urinary tract infections

## Abstract

Urinary tract infections are one of the most common infectious diseases, with *Escherichia coli* as the predominant pathogen. Traditional diagnostic methods fail to meet clinical demands for rapid and specific detection. Here, we developed an efficient urine *E. coli* detection strategy via a reporter phage cocktail. Four reporter phages (T2::*Nluc*, T4::*Nluc*, T5::*Nluc*, T6::*Nluc*) were constructed by the CRISPR/Cas9 system combined with homologous recombination. One-step growth curves, optimal multiplicity of infection, and lytic efficiency showed that the *Nluc* gene block insertion exerted heterogeneous effects on phages. Luminescence assays demonstrated that all five reporter phages (including previously preserved T7::*Nluc*) and the cocktail offered favorable limits of detection (≥10^3^ CFU/mL), high specificity, and no urine matrix interference. However, single phages exhibited limited coverage among 177 clinical *E. coli* isolates. But the reporter phage cocktail remedies this limitation. In large-scale clinical validation, the cocktail achieved sensitivity 73.15% (63.76%–81.22%), specificity 100.00% (99.53%–100.00%), positive predictive value (PPV) 100.00% (95.44%–100.00%), and negative predictive value (NPV) 96.42% (95.18%–97.36%) (all 95% confidence interval [CI]), and excellent concordance with the gold-standard method (Kappa = 0.83, 95% CI: 0.77–0.89), greatly outperforming single reporter phages (~40.00% sensitivity). This method requires no sample pretreatment, is simple to operate, and completes detection within 4 h, significantly improving diagnostic efficiency. Accordingly, it provides a novel platform for pathogen detection and supports the clinical translation of reporter phage diagnostics.

**IMPORTANCE:**

Urinary tract infections impose substantial economic and public health burdens. In this study, we successfully constructed *Escherichia coli*-specific reporter phages T2::*Nluc*, T4::*Nluc*, T5::*Nluc*, and T6::*Nluc*. Combined with the previously preserved T7::*Nluc*, these phages formed a reporter phage cocktail. Co-cultivation of this cocktail with clinical samples enabled rapid and specific detection of *E. coli* in clinical urine, with a significantly shortened detection time (4 h) and good concordance with the gold-standard detection method (Kappa = 0.83), effectively improving detection efficiency and accuracy. This novel pathogen detection platform, integrating specific recognition and signal amplification, not only provides a new technical approach for the rapid and accurate diagnosis of clinical urinary tract infections but also effectively promotes the coordinated improvement of infectious disease diagnosis and treatment in terms of timeliness-precision-cost.

## INTRODUCTION

Urinary tract infections (UTIs) rank among the most prevalent infectious diseases worldwide (1), affecting 405 million individuals annually and leading to approximately 240,000 deaths each year (2, 3). Notably, the global mortality attributable to UTIs has surged by 140% from 1990 to 2019 (3), imposing a severe burden on global public health and incurring substantial economic losses. UTIs are commonly caused by a diverse range of pathogens, among which *Escherichia coli* is the predominant etiologic agent (4).

At present, urine culture remains the gold standard for the diagnosis of UTIs (5); however, this method suffers from prolonged turnaround time (24–48 h), an increased risk of missed detection of fastidious bacteria, and infrequent reporting of polymicrobial infections (5, 6). Emerging molecular biological technologies, including polymerase chain reaction (PCR), enzyme-linked immunosorbent assay, metagenomic next-generation sequencing, and targeted next-generation sequencing, are associated with stringent requirements for technical expertise and instrumentation, while failing to discriminate between viable and non-viable bacteria and entailing considerable costs (3, 7, 8). Accurate identification of pathogenic bacteria constitutes a core prerequisite for clinical decision-making in the diagnosis and treatment of infectious diseases. Furthermore, the continuous spread of bacterial antimicrobial resistance has created an urgent clinical need for the development of cost-effective, rapid, sensitive, accurate, and specific detection methods for pathogenic bacteria.

Bacteriophages are natural viruses that can selectively infect specific bacteria at the genus, species, or strain level (9–11). In recent years, a series of high-efficiency diagnostic tools for pathogenic bacteria based on phages have been developed, which possess the 4S properties (simplicity, speed, sensitivity, and specificity) (12). Among these, reporter phages are recognized as a clinical detection element with great application potential for pathogenic bacteria identification. Specifically, reporter genes (e.g., *GFP*, *Nluc*, *ALP*, *β-gal*) are integrated into the phage genome via genetic engineering to construct reporter phages (13, 14). Upon infection of host bacteria, the reporter proteins are expressed to enable signal visualization. Meanwhile, the proliferation of reporter phages generates a large number of progeny phages, which endows the system with the advantage of signal self-amplification, thus achieving sensitive and specific detection of pathogenic bacteria. Furthermore, reporter phages exhibit a short detection time, facile production, low cost, and high stability against temperature and pH fluctuations (15), thus serving as an ideal tool for the identification of pathogenic bacteria.

However, clinical pathogens display prominent clonal diversity (16). Carter C’s team recovered 199 clinical *E. coli* isolates from community- and hospital-acquired UTIs that encompassed 70 distinct sequence types (STs) (17). ST1193, ST131, ST405, and ST38 are dominant uropathogenic *E. coli* lineages and exhibit strong associations with high-level antimicrobial resistance (18–20). In addition, *E. coli* strains belonging to different STs vary in surface antigens, antimicrobial resistance patterns, and epidemiological transmission features (21), with divergent O serotypes, fimbrial types, and capsules across subclones (22). Nevertheless, the strict host specificity of single phages leads to severely limited host coverage, which fails to meet the detection and therapeutic requirements of clinically complex polyclonal samples and constitutes a major bottleneck restricting their clinical translation. Against this background, a phage cocktail consisting of multiple phages with complementary host ranges can substantially broaden total host coverage by leveraging the divergent host receptor-binding properties of individual phages (23, 24), thereby overcoming this critical barrier to clinical translation.

In this study, we engineered a panel of *E. coli*-specific reporter phages (T2::*Nluc*, T4::*Nluc*, T5::*Nluc*, and T6::*Nluc*) via the CRISPR/Cas9 system combined with homologous recombination technology, with T7::*Nluc* generated in our previous research. These phages will be formulated into a reporter phage cocktail, enabling this technique to serve as a highly specific, rapid adjunctive diagnostic tool for urinary tract infections caused by *E. coli*. This strategy is anticipated to enable timely optimization of clinical therapeutic regimens and ultimately alleviate the substantial public health and economic burdens stemming from drug-resistant infections and delayed clinical intervention.

## MATERIALS AND METHODS

### Phages, strains, and plasmids

Phages T2, T4, T5, T6, T7, and the *E. coli* strain BL21 were purchased from the American Type Culture Collection. A total of 177 clinical *E. coli* isolates from urine samples and one strain each of other pathogenic bacteria (*Enterococcus faecalis*, *Enterococcus faecium*, *Klebsiella pneumoniae*, *Acinetobacter baumannii*, *Pseudomonas aeruginosa*, *Staphylococcus aureus*, *Staphylococcus epidermidis*, and *Staphylococcus hominis*) were obtained from the bacterial strain repository of the Department of Clinical Laboratory Medicine, The First Medical Center of PLA General Hospital. All clinical strains were identified by matrix-assisted laser desorption/ionization time-of-flight mass spectrometry (MALDI-TOF MS). Among all isolates, 177 *E. coli* strains were collected and stored from urine samples of distinct patients during January 2011 to November 2015 and January 2018 to March 2023, after removal of duplicates. Other non-*E*. *coli* strains were isolated and identified from clinical urine samples from December 2024 to January 2025. The pCRISPR and pCas9 plasmids were purchased from Hunan Fenghui Biotechnology Co., Ltd. This study was approved by the Ethics Committee of Chinese PLA General Hospital, with the approval number S2023-580-03.

### Construction of homologous recombination plasmids via Gibson assembly

Non-essential genes of each phage were selected as genetic engineering loci. *soc* and *hoc* are recognized as non-essential genes of phage T4 (25), and *soc* is also dispensable in phage T2 (26). Although studies on phage T6 remain relatively limited, the genome annotation from the National Center for Biotechnology Information designates the product of gene locus *KMB99_gp220* as a “Hoc-like head decoration” protein. Given that the genomic similarity among phages T2, T4, and T6 exceeds 96% (27), *soc* of T2 and T4, *KMB99_gp220* of T6 (encoding a Hoc-like head decoration protein) were chosen as the integration loci for the *Nluc* gene. For phage T5, the non-essential gene *dmp* was selected as the site for genetic modification (28). Nucleotide sequences of approximately 1,000 bp flanking each of the aforementioned non-essential genes were amplified as the upstream and downstream homologous arms; the *Nluc* gene block, preserved from the preliminary work of our team (14), contained the T7 promoter, ribosome binding site, pelB leader sequence, and *Nluc* gene. Primers with 20 bp overlapping sequences at both ends were designed for each of the above sequences (Table S1) and synthesized by Sangon Biotech (Shanghai) Co., Ltd.

Homologous arms and *Nluc* gene block fragments were PCR-amplified and recovered, then assembled into homologous recombination plasmids via Gibson assembly using the ClonExpress Ultra One Step Cloning Kit (Vazyme, China). The recombinant plasmids were transformed into BL21(DE3) competent cells, with verification by colony PCR.

### Construction of CRISPR/Cas9 system

Single-guide RNA (sgRNA) targeting the aforementioned non-essential genes of phage T2, T5, and T6 were designed via the online web tool (sgRNAcas9-AI, https://github.com/muxiaoxiong/sgRNAcas9-AI), with the top 10 sgRNAs ranked by predicted activity selected. The sgRNA targeting the *soc* gene of phage T4 was soc-cr6, which exhibits excellent cleavage efficiency as reported by the Duong MM research group (29). *BsaI* restriction sites were added to both ends of all sgRNA sequences, which were then synthesized by Sangon Biotech (Shanghai) Co., Ltd. (Table S2). Then, the sgRNA oligonucleotides were phosphorylated and annealed to form double-stranded DNA using T4 Polynucleotide Kinase (New England Biolabs, USA). The resulting fragments were ligated with linearized pCRISPR plasmids (digested with *BsaI* overnight) via T4 DNA Ligase (New England Biolabs, USA), then transformed into BL21(DE3) competent cells. Positive clones were validated by colony PCR and Sanger sequencing.

pCRISPR-sgRNA plasmids were extracted using the FastPure Plasmid Mini Kit (Vazyme, China). The pCas9 and pCRISPR-sgRNA plasmids were sequentially transformed into BL21(DE3) competent cells to construct the CRISPR/Cas9 system.

The actual cleavage efficiency of sgRNA was determined by the efficiency of plating (EOP), where a lower EOP indicates higher cleavage efficiency of the corresponding sgRNA. EOP was calculated as the ratio of phage plaque-forming units on pCRISPR-sgRNA-pCas9-BL21 double-layer agar plate to those on pCRISPR-pCas9-BL21 double-layer agar plate. For the double-layer agar assay (30), 200 μL of log-phase pCRISPR-sgRNA-pCas9-BL21 or pCRISPR-pCas9-BL21 was mixed with 100 μL of the corresponding phage at an appropriate titer in molten top agar (LB + 0.625% [wt/vol] agar), supplemented with kanamycin sulfate (50 μg/mL) and chloramphenicol (30 μg/mL). The mixture was immediately poured onto LB solid agar plates and incubated overnight at 37°C for plaque formation.

### Construction of reporter phages by CRISPR/Cas9-mediated homologous recombination

**Fig 1:**
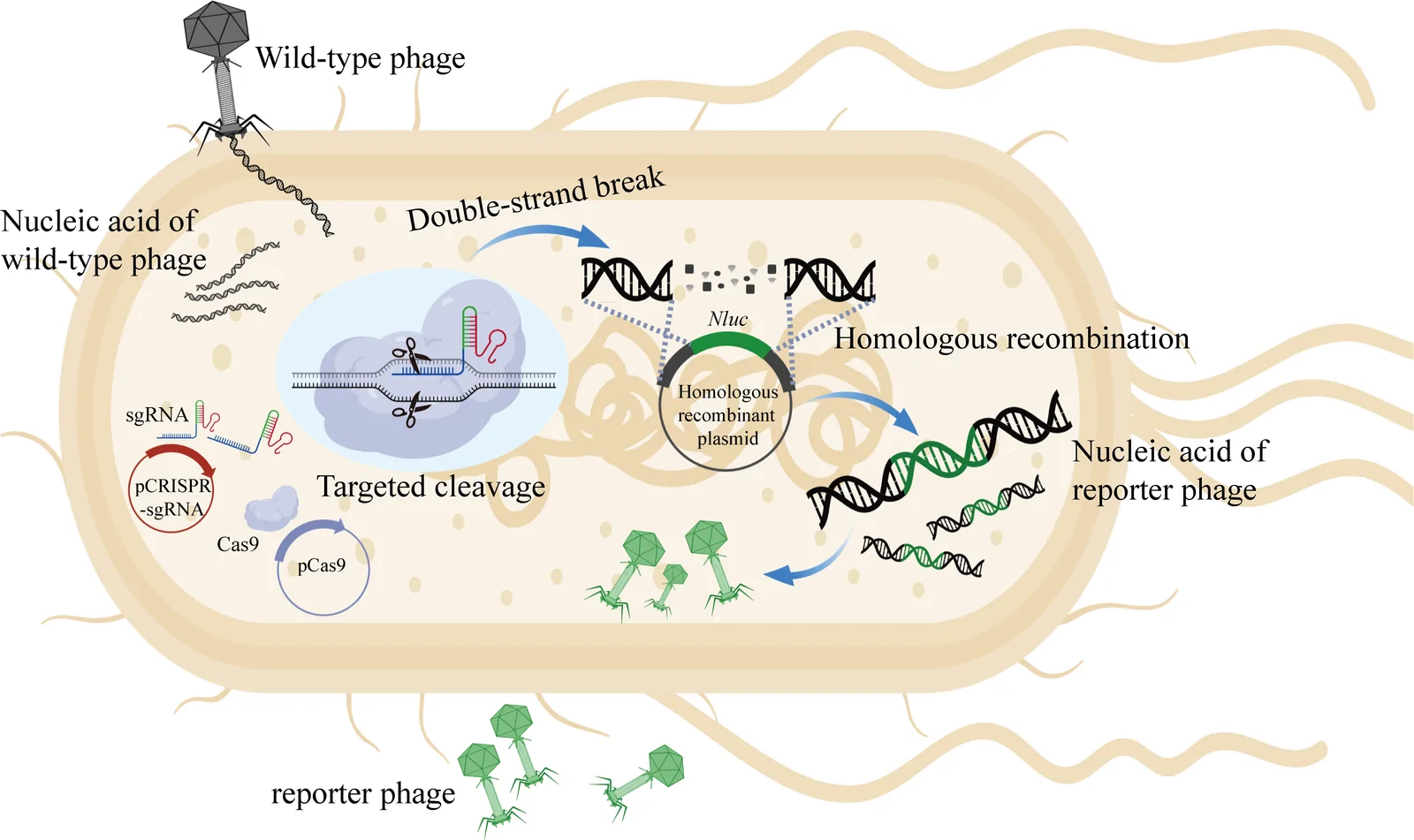
Schematic of CRISPR/Cas9-mediated homologous recombination.

Classical homologous recombination exhibits extremely low efficiency (10⁻¹⁰ to 10⁻⁴) for phage gene editing (31, 32). In our previous study, the CRISPR/Cas9 system combined with homologous recombination technology significantly improved the construction and screening efficiency of reporter phage to 87.5% (21/24 recombination success rate) (14), and this strategy was therefore adopted in this study for reporter phage generation. Homologous recombination plasmids were individually transformed into the corresponding pCRISPR-sgRNA-pCas9-BL21 with the highest cleavage efficiency to construct triple-plasmid BL21. The triple-plasmid BL21 was infected with the corresponding phage by the double-layer agar assay. During this process, the CRISPR/Cas9 system, guided by sgRNA, targeted and cleaved the genome of the corresponding wild-type phage, generating massive double-strand breaks that significantly increased homologous recombination efficiency. Meanwhile, the CRISPR/Cas9 system exerted a counter-selection effect (11, 32), enriching recombinant reporter phages in the progeny and facilitating their screening (Fig. 1).

The substrate was prepared using the Nano-Glo Luciferase Assay Kit (Promega, USA), and the principle is as follows:


$$\text{ Furimazine }+O_{2}\overset{\text{ Nanoluciferase }}{\to }\text{ Furimamide }+{CO}_{2}+\text{ Light }\left({Em}_{\max}=460\;nm\right).$$

The substrate was added onto the plaques on double-layer agar plates. A gel imaging system equipped with Image Lab 6.1 software (Bio-Rad, USA) was used for detection. The “Blot-Chemi” program was applied with an exposure time of 5 s to identify reporter phages, which appeared as black plaques on the plate (30). In parallel, the “Blot-Colorimetric” program with automatically optimized exposure was used to capture the total plaque image. Reporter phages were screened by overlaying the two images.

Phages from the black plaques were collected and co-cultured with the corresponding optimal pCRISPR-sgRNA-pCas9-BL21 for approximately 5 h. Wild-type phage co-cultured with BL21 served as a control. A 100 μL aliquot of each co-culture was transferred to a 96-well plate, followed by the addition of 20 μL substrate. After incubation at room temperature for 3 min, luminescence signals (relative light units, RLU) were measured using the Infinite M200 PRO microplate reader (Tecan, Switzerland) with Tecan i-control 2.0.10.0 software.

Furthermore, phages from black plaques were mixed with 10 mM EDTA solution, incubated at 65°C for 10 min, and then cooled to 4°C. The treated samples were used as templates for phage PCR to identify the recombined reporter phages T2::*Nluc*, T4::*Nluc*, T5::*Nluc*, and T6::*Nluc*. The selected reporter phages were further verified by Sanger sequencing.

### Comparison of biological characteristics

#### One-step growth curves

Wild-type or reporter phage (5 mL at 1 × 10⁷ PFU/mL) was combined with 5 mL of BL21 culture at 1 × 10⁸ CFU/mL. After incubation in a 37°C water bath for 10 min, the mixture was centrifuged at 5,000 × *g* for 10 min. The supernatant was discarded, and the pellet was washed with 1× PBS buffer, followed by a second centrifugation and supernatant removal (33, 34). The pellet was resuspended in 10 mL of LB medium and cultured at 37°C with shaking at 200 rpm for 120 min. Samples were collected every 10 min, filtered through a 0.22 μm syringe filter, and the phage titer was determined using the double-layer agar assay. Experiments were conducted with three biological and three technical replicates. Burst size = number of phages released at the end of the burst period/number of infected host cells at the early latent period (35).

#### The optimal multiplicity of infection

Wild-type or corresponding reporter phages with different titers were mixed with BL21 at 1 × 10⁸ CFU/mL at various multiplicities of infection (MOI). After incubation at 37°C with shaking at 200 rpm for 6 h, the mixtures were filtered through a 0.22 μm syringe filter, and phage titers were determined by the double-layer agar assay. Each group was performed in triplicate. The experiment included three biological and three technical replicates. The MOI corresponding to the highest phage titer was defined as the optimal MOI (34).

#### The lysis efficiency

BL21 was adjusted to 1 × 10⁸ CFU/mL. At an MOI of 0.1, four groups were set up: “BL21 + LB,” “BL21 + wild-type phage,” “BL21 + reporter phage,” and “LB.” All groups were cultured at 37°C with shaking at 200 rpm for 6 h. From 0 min onward, 100 μL of the culture was collected every 10 min into a 96-well plate, and the OD_600nm_ was measured. Lysis curves were plotted with time as the x-axis and OD_600nm_ as the y-axis. All experiments were carried out in triplicate at both the biological and technical levels.

### Limit of detection of reporter phages

Overnight-cultured BL21 was serially diluted 10-fold to concentrations ranging from 1 × 10⁶ CFU/mL to 1 × 10° CFU/mL. A 5.4 mL aliquot of the serially diluted BL21 was co-cultured with reporter phages at an appropriate titer (background luminescence of approximately 10 RLU) at 37°C with shaking at 200 rpm. A group containing only reporter phages served as the negative control. At 10 min intervals, 100 μL of sample was removed, mixed with substrate, and the luminescence signal was measured. Time-luminescence curves were plotted with time as the x-axis and luminescence as the y-axis. The positivity threshold was defined as the mean background luminescence plus three times the standard deviation (11). The limit of detection (LOD) was determined as the lowest bacterial concentration that produced a luminescence signal above the threshold. Each experiment consisted of three technical replicates and was independently repeated three times.

### The influence of urine matrix on the detection

Clean midstream urine samples were collected from the Department of Clinical Laboratory Medicine, The First Medical Center of PLA General Hospital, and filtered through a 0.22 μm syringe filter (30). The filtered urine was mixed with *E. coli* to achieve urine matrix ratios of 0%, 20%, 40%, 60%, 80%, and 100%, while maintaining a consistent *E. coli* concentration of approximately 1 × 10⁶ CFU/mL across all groups. A group containing only LB medium served as the negative control. Appropriate reporter phages were added to each group, followed by incubation at 37°C with shaking at 200 rpm. Every 10 min, 100 μL of the co-culture was removed, mixed with substrate, and subjected to luminescence measurement. Three biological replicates and three technical replicates were performed.

### Detection specificity of reporter phages

One strain each of *E. faecalis*, *E. faecium*, *K. pneumoniae*, *A. baumannii*, *P. aeruginosa*, *S. aureus*, *S. epidermidis*, *S. hominis*, and BL21 (positive control) was cultured overnight at 37°C with shaking at 200 rpm. Each bacterial suspension was diluted 100-fold and further co-cultured with reporter phages. During which, 100 μL of the co-culture was collected every 10 min, mixed with substrate, and the luminescence was determined. A group containing only reporter phage was set as the negative control. The experiment included three biological and three technical replicates.

### Sucrose density gradient centrifugation of reporter phages

A 20-mL volume of 30% sucrose solution in PBS was added to an ultracentrifuge tube, followed by 18 mL of reporter phage. After centrifugation at 26,000 rpm for 3 h at 4°C, the supernatant was carefully removed. The tube was inverted and air-dried, and the phage pellet was resuspended in 1 mL of 1× PBS. The titer and luminescence were determined, and the reporter phages were stored at 4°C.

### Detection coverage of reporter phages among clinical isolates

Whole-genome sequencing of all 177 *E. coli* isolates was performed on the Illumina NovaSeq platform (Illumina, USA). The sequencing depth ranged from 187× to 464×, with an average depth of approximately 289×. For genome assembly metrics, the total genome length varied between 4.5 and 5.5 Mb; the contig N50 values spanned 78 to 659 kb (median, 195 kb), and the average GC content was 50.60%. The multi-locus sequence typing (MLST) analysis and serotype prediction were conducted via the MLST and Serotype Finder software. The phylogenetic tree was constructed by kSNP4 (v4.0) based on single-nucleotide polymorphisms. Visualization was performed with the Interactive Tree Of Life (iTOL, v6) to ensure the clonal diversity and representativeness of clinical epidemic typing of *E. coli*.

The highest titer of each reporter phage yielding a background luminescence of approximately 10 RLU after sucrose density gradient centrifugation was defined as its clinical working concentration. All 177 *E. coli* isolates were cultured overnight at 37°C with shaking at 200 rpm. After 100-fold dilution (final bacterial concentration, ~10⁶ CFU/mL), each isolate was co-incubated with individual reporter phages or the cocktail for 4 h, and then subjected to luminescence measurement. LB broth and BL21 co-cultured with the reporter phages or cocktail were set as negative and positive controls, respectively. All assays were performed in three technical replicates.

### Preparation and performance evaluation of the reporter phage cocktail

T2::*Nluc*, T4::*Nluc*, T5::*Nluc*, T6::*Nluc*, and T7::*Nluc* were mixed at a 1:1:1:1:1 ratio to prepare the phage cocktail, with each phage at 10 times its clinical working concentration. The LOD and detection coverage of the cocktail were determined as previously described (the final concentration of each phage in the cocktail was 1/5 of its clinical working concentration).

Validation of the LOD against clinical isolates. Strains identifiable by T2::*Nluc*, T4::*Nluc*, T5::*Nluc*, T6::*Nluc*, T7::*Nluc,* and the reporter phage cocktail were selected from 177 clinical *E. coli* isolates. BL21 was set as the positive control, while LB broth was used as the negative control. Overnight bacterial cultures were diluted to 10⁴ CFU/mL and co-cultured with single reporter phages or the reporter phage cocktail at 37°C, 200 rpm for 4 h prior to luminescence detection. Three biological and technical replicates were conducted for all assays.

### Validation with clinical urine samples

All fresh clinical urine specimens for urine culture were collected daily without bias at the Department of Clinical Laboratory Medicine, The First Medical Center of PLA General Hospital during two periods: December 2024 to January 2025, and July 2025 to December 2025. The infection process of reporter phages against pathogenic bacteria is barely affected by antibiotic resistance (36–38); the antibiotic treatment history of patients was not considered during specimen collection.

Urine samples were processed immediately without storage and subjected to standard urine culture, followed by species identification via MALDI-TOF MS. Urine culture positivity was defined as the growth of a single bacterial species at ≥10⁵ CFU/mL (5). Meanwhile, 900 μL urine was mixed with 100 μL reporter phage or cocktail to achieve working concentration, incubated at 37°C, 200 rpm for 4 h, and supplemented with substrate for luminescence measurement. Three technical replicates were conducted to detect *E. coli* infection. Comparisons between the two methods were performed to obtain various diagnostic parameters, including sensitivity and specificity.

### Statistical analysis

Figures were generated using BioGDP (https://biogdp.com/) (39), OriginPro 2025, GraphPad Prism 8.0.2, and Adobe Illustrator 2024. Statistical analyses were performed using SPSS 27.0.1, GraphPad Prism 8.0.2, and MedCalc v23.4.9. Repeated measures ANOVA assessed differences in one-step growth curves. Changes in luminescence of reporter phages before and after sucrose density gradient centrifugation were analyzed using multiple t-tests. The Kruskal-Wallis H test was used to compare phage lysis efficiency and intergroup differences across different proportions of urine matrix. The detection coverage of the individual reporter phages and the reporter phage cocktail among 177 clinical strains was compared using a two-sided Fisher’s exact test. *P* < 0.05 was considered statistically significant. Diagnostic performance metrics, including sensitivity, specificity, positive predictive value (PPV), negative predictive value (NPV), accuracy, and Cohen’s kappa, along with their respective 95% confidence intervals (CIs), were calculated using MedCalc v23.4.9.

## RESULTS

### Construction of reporter phages

Colony PCR and Sanger sequencing confirmed that all homologous recombination plasmids and pCRISPR-sgRNA plasmids were successfully constructed, except for the homologous recombination plasmid of T2 (Fig. S1 to S3). This homologous recombination plasmid lacked the upstream homology arm, probably due to the presence of numerous 4–8 bp palindromic sequences, long single-base repeats, and local high GC-rich regions in the upstream homology arm, which tend to form stable secondary structures and drastically reduce the efficiency of specific recombination. In addition, the linearized pCE-Zero vector in the ClonExpress Ultra One Step Cloning Kit contained a T7 promoter sequence at the junction with the upstream homology arm, and the starting segment of the *Nluc* gene block also contained the T7 promoter, which facilitated non-specific recombination. Thus, direct ligation occurred during assembly, leading to the eventual loss of the upstream homology arm. The upstream homology arms of other phages showed no obvious secondary structure features and did not affect Gibson assembly of the homologous recombination plasmids. Using linearized pCE-Zero vector as the template, primers T2-vector-F/R were designed to amplify the T7 promoter-deleted linearized pCE-Zero vector by PCR (Fig. S2A). The amplified product was recovered, Gibson assembly and verification of the homologous recombination plasmid were performed again (Fig. S2B).

**Fig 2:**
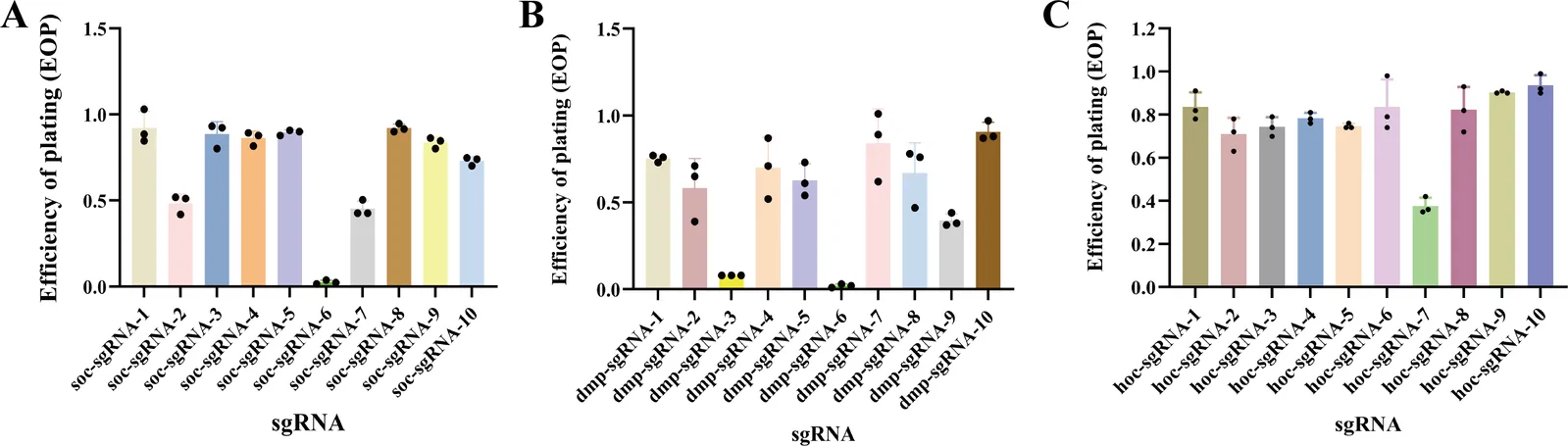
Determination of the actual cleavage efficiency of sgRNAs. (**A**) Cleavage efficiency of 10 sgRNAs targeting the *soc* gene of T2. (**B**) Cleavage efficiency of 10 sgRNAs targeting the *dmp* gene of T5. (**C**) Cleavage efficiency of 10 sgRNAs targeting the *KMB99_gp220* gene of T6.

In terms of EOP, T2-soc-sgRNA-6, T5-dmp-sgRNA-6, and T6-hoc-sgRNA-7 exhibited the highest actual cleavage efficiency (Fig. 2). Accordingly, the homologous recombination plasmids of T2, T4, T5, and T6 were transformed into pCas9-pCRISPR-soc-sgRNA-6-BL21, pCas9-pCRISPR-soc-cr-6-BL21, pCas9-pCRISPR-dmp-sgRNA-6-BL21, and pCas9-pCRISPR-hoc-sgRNA-7-BL21, respectively, to construct the triple-plasmid strains.

**Fig 3:**
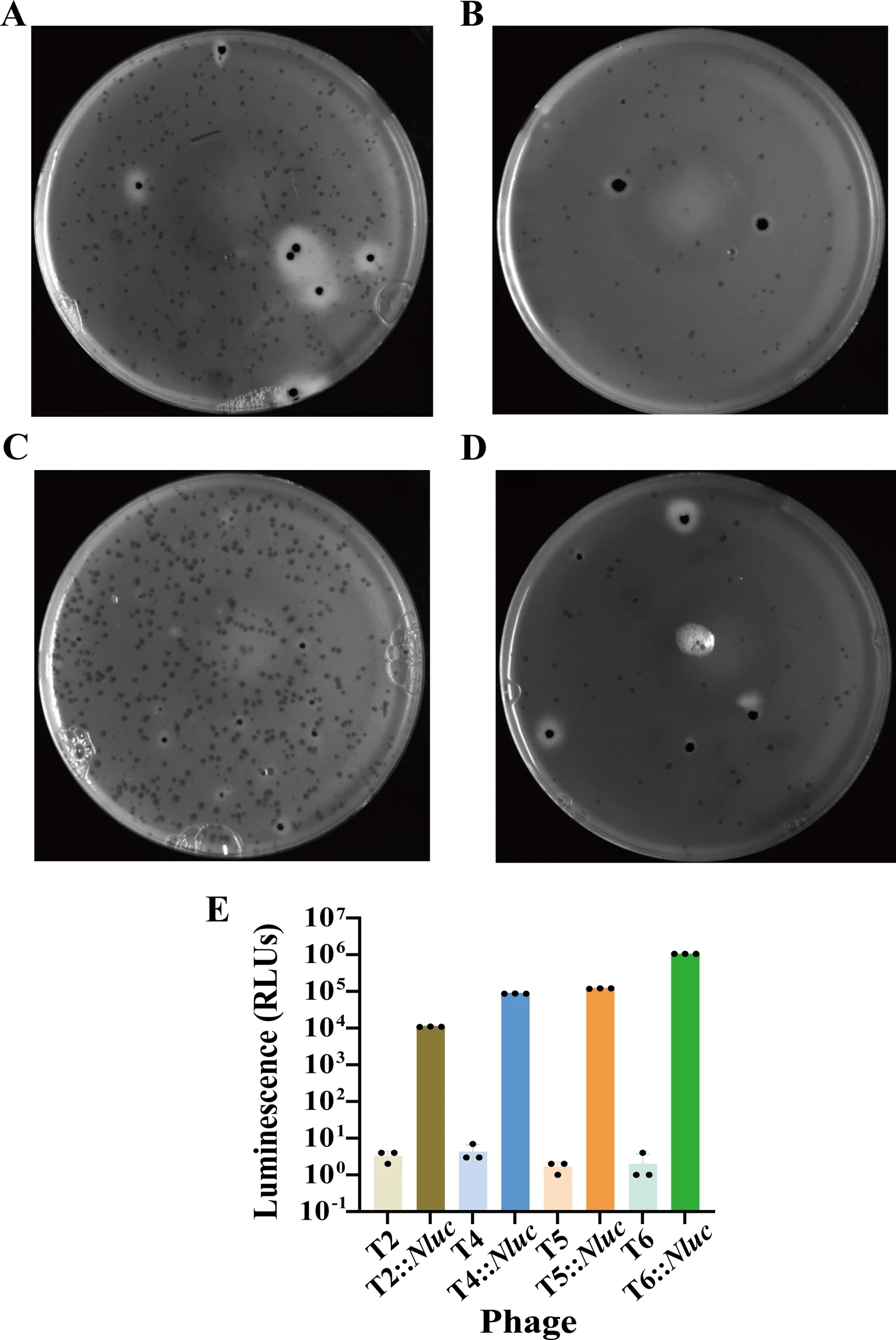
Screening and luminescence function verification of reporter phages. (**A–D**) Screening of T2::*Nluc*, T4::*Nluc*, T5::*Nluc*, and T6::*Nluc* reporter phages. Black plaques indicate those formed by reporter phages. (**E**) Luminescence function verification of reporter phages.

Each phage was used to infect the corresponding triple-plasmid BL21 via the double-layer agar assay, and CRISPR/Cas9-mediated homologous recombination was performed to generate reporter phages (Fig. 1). Reporter phages with luminescence activity were initially screened by plaque selection and luminescence detection (Fig. 3). Then, PCR and Sanger sequencing further verified the successful construction of the reporter phages (Fig. S4).

### Comparison of biological characteristics

Biological characteristics of phages are summarized in Table 1. The optimal MOI of T2::*Nluc* was 0.00001, which was lower than that of T2 (Fig. S5), and its burst size was 3.5 times that of T2 (*P* < 0.05). T4::*Nluc* displayed an optimal MOI of 0.1, which was lower than that of T4 (1), while no significant difference in burst size was noted between them (*P* > 0.05) (Fig. S6). For T5::*Nluc*, the optimal MOI was 1 (higher than T5’s 0.1), and its burst size was 12.3 times that of T5 (*P* < 0.05) (Fig. S7). T6::*Nluc* exhibited an optimal MOI of 0.01, lower than that of T6 (0.1), and its burst size was significantly reduced compared to T6 (*P* < 0.05) (Fig. S8). Furthermore, lytic efficiency showed no significant differences between T2::*Nluc*, T6::*Nluc*, and their respective wild-type phages (*P* > 0.05), while T4::*Nluc* and T5::*Nluc* differed significantly from their wild-type counterparts (*P* < 0.05). Specifically, the overall OD_600nm_ of the “BL21 + T4::*Nluc*” was slightly higher than that of the “BL21 + T4” group (Fig. S6), indicating a moderately lower lytic activity of T4::*Nluc*. And the inflection point at which OD_600nm_ started to decline was delayed by 40 min in the “BL21 + T5::*Nluc*” group compared with the “BL21 + T5” (Fig. S7).

**TABLE 1:** Biological characteristics of phages^*a*^

Phage	Latent period (min)	Lysis period (min)	Burst size (PFU/mL)	The optimal MOI
T2	10	10–80	32.24	0.0001
T2::*Nluc*	10	10–80	112.89	0.00001
T4	10	10–50	21.04	1
T4::*Nluc*	10	10–50	17.50	0.1
T5	10	10–100	103.96	0.1
T5::*Nluc*	10	10–100	1,279.49	1
T6	10	10–40	185.51	0.1
T6::*Nluc*	10	10–40	16.52	0.01

^*a*^ MOI, multiplicity of infection.

### Evaluation of reporter phage detection performance

#### The limit of detection

**Fig 4:**
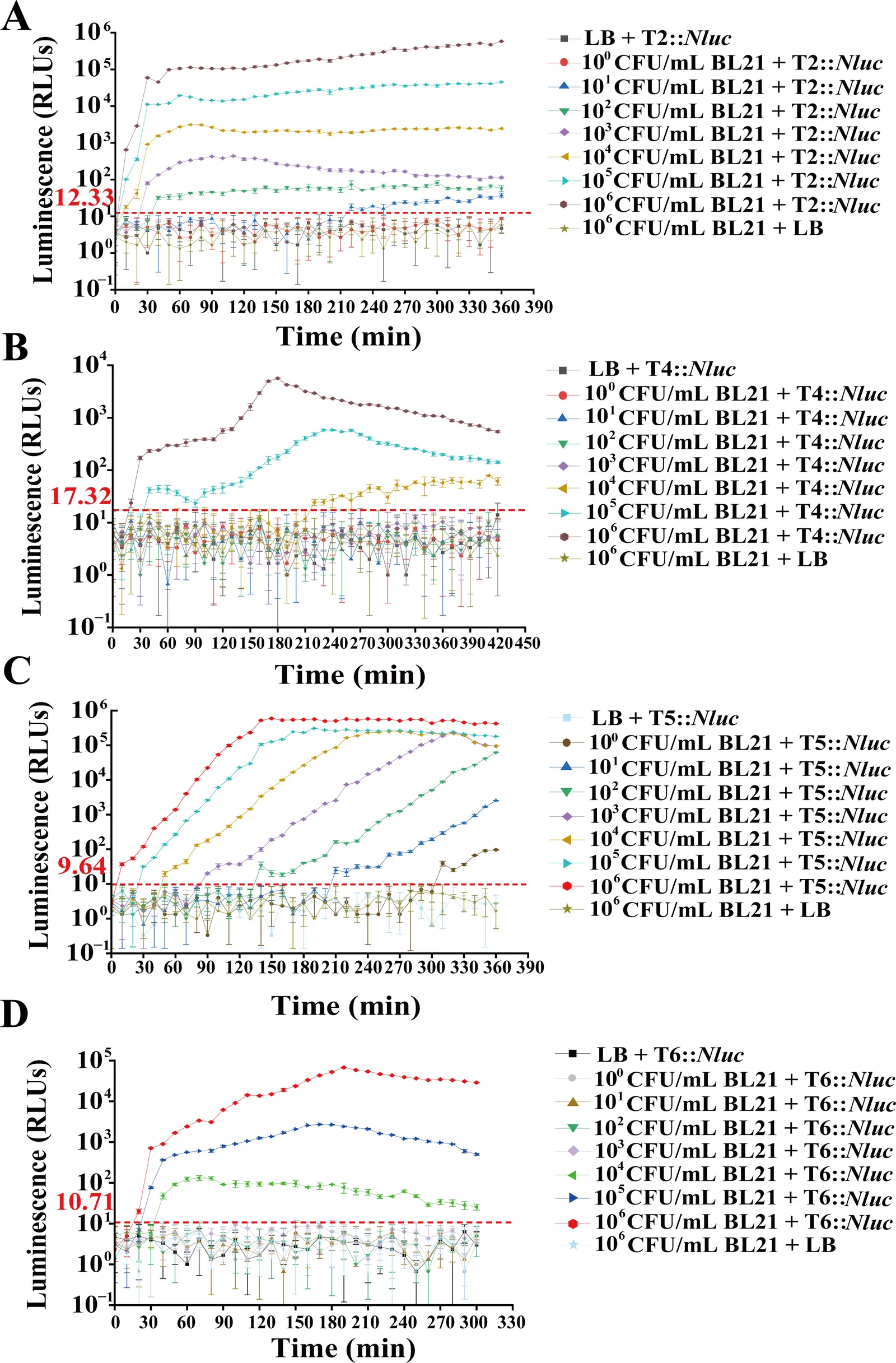
Limit of detection for reporter phages. (**A**) LOD for T2::*Nluc*. The positivity threshold was defined as 12.33 RLU. BL21 at a concentration as low as 10¹ CFU/mL was detected within 220 min. (**B**) LOD for T4::*Nluc*. Using a positivity threshold of 17.32 RLU, BL21 at ≥10⁴ CFU/mL was detected by 220 min. (**C**) LOD for T5::*Nluc*. With a positivity threshold set at 9.64 RLU, the assay detected BL21 at concentrations below 10 CFU/mL within 310 min. (**D**) LOD for T6::*Nluc*. At a positivity threshold of 10.71 RLU, BL21 at ≥10⁴ CFU/mL was detected within 40 min. LOD, limit of detection.

Following co-culture of the reporter phages with serially diluted BL21 ranging from 1 × 10⁶ to 1 × 10° CFU/mL, time-luminescence curves demonstrated substantial disparities in the detection efficiency of the reporter phages. T2::*Nluc* enabled detection of BL21 at concentrations ≥10¹ CFU/mL within 220 min. Both T4::*Nluc* and T6::*Nluc* displayed a LOD of 10⁴ CFU/mL; however, T6::*Nluc* yielded a detectable signal within 40 min, markedly earlier than the 220 min required for T4::*Nluc*. Notably, T5::*Nluc* and T7::*Nluc* exhibited significantly superior detection sensitivity, achieving reliable identification of *E. coli* at 10 CFU/mL or below, with corresponding detection durations of 310 and 300 min, respectively (Fig. 4). The LOD for T7::*Nluc* was previously verified in our earlier research (14).

#### The influence of urine matrix

**Fig 5:**
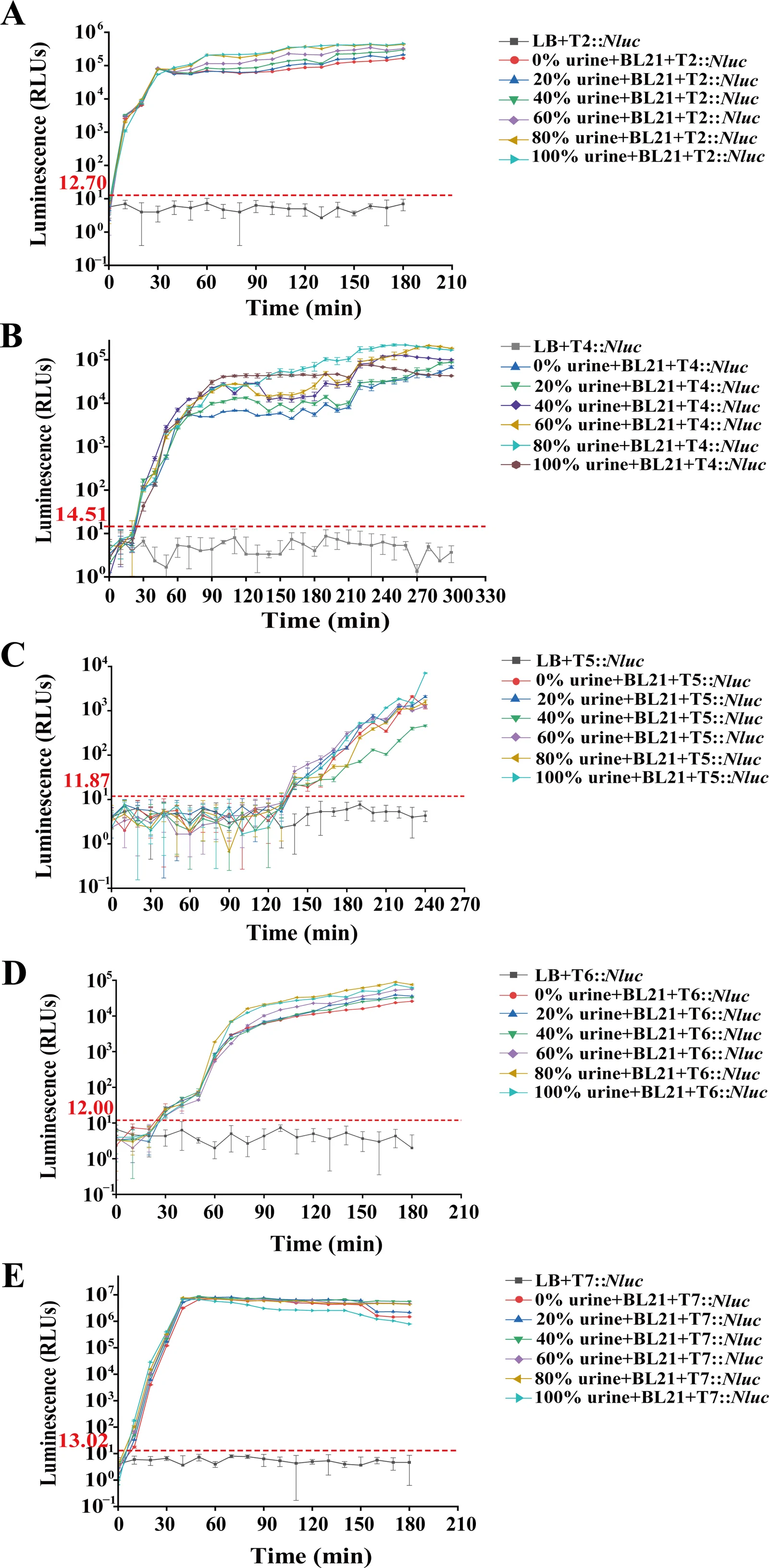
Urine matrix influence on reporter phage detection performance. (**A–E**) Influence of urine matrix on the detection performance of T2::*Nluc*, T4::*Nluc*, T5::*Nluc*, T6::*Nluc*, and T7::*Nluc*, respectively. After the addition of the same reporter phage, positive luminescence signals were observed at identical times across all urine matrix proportion groups, with no significant differences detected (Kruskal-Wallis H test, *P* > 0.05).

To evaluate the effect of urine matrix on reporter phages’ performance, varying urine concentration groups (0%, 20%, 40%, 60%, 80%, and 100%) and a negative control were established for each reporter phage, and the luminescence of each group was monitored over time. As shown in Fig. 5, no statistically significant differences were observed among groups with different urine proportions for the same reporter phage (*P* > 0.05), and positive luminescence signals emerged at identical times across all groups. These findings demonstrate that urine matrix does not interfere with the interaction between reporter phages and *E. coli*, nor compromise the luminescent reaction of Nluc protein. Consequently, sample pretreatment is dispensable, which greatly streamlines the detection workflow for clinical specimens.

#### Detection specificity

**Fig 6:**
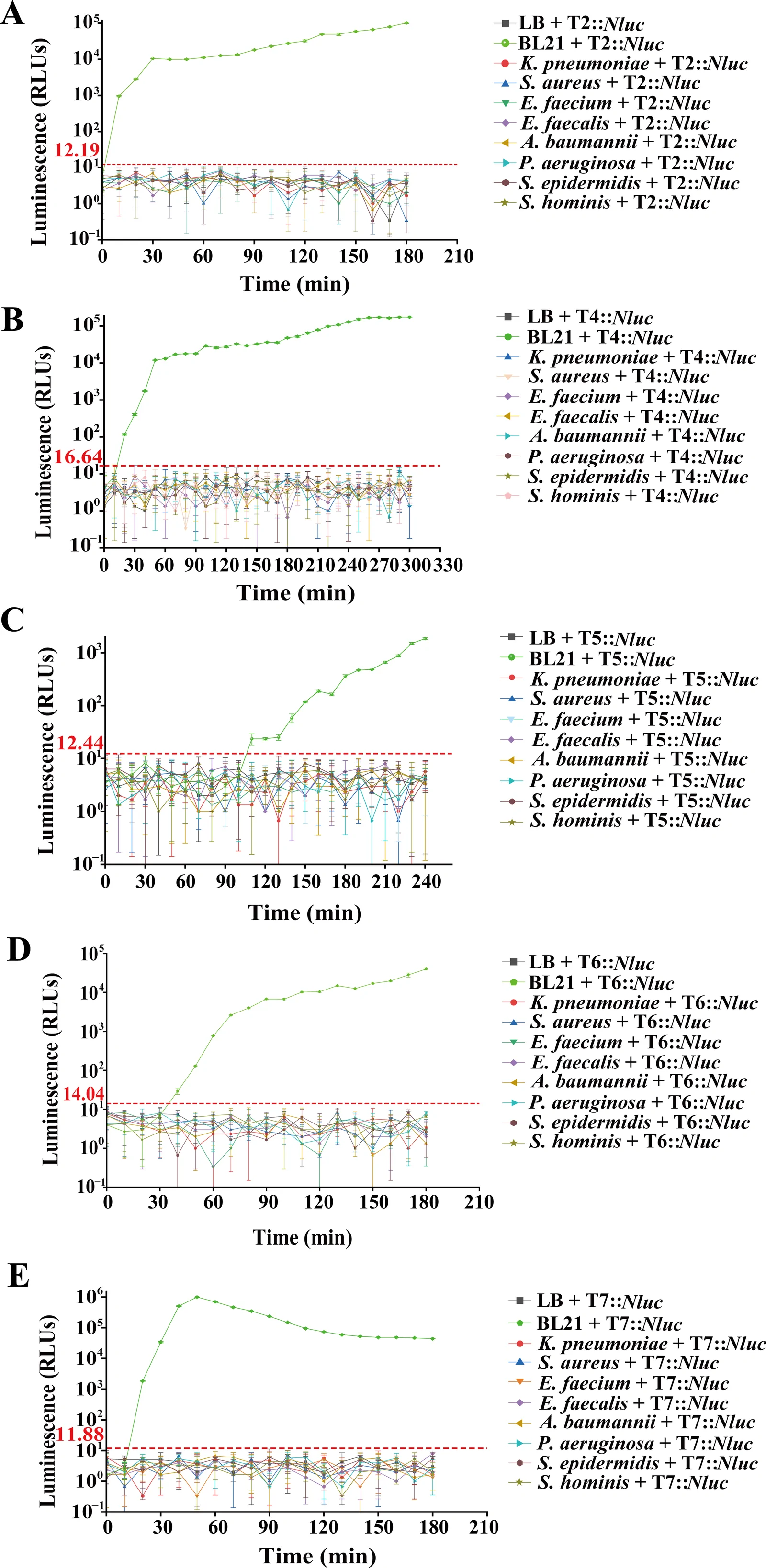
Detection specificity of reporter phages. (**A–E**) Detection specificity of T2::*Nluc*, T4::*Nluc*, T5::*Nluc*, T6::*Nluc*, and T7::*Nluc* reporter phages against common uropathogenic bacteria.

UTIs are commonly caused by a variety of pathogenic bacteria. Time-luminescence curves were recorded following co-culture of clinically predominant pathogens with different reporter phages. The results demonstrated that T2::*Nluc*, T4::*Nluc*, T5::*Nluc*, T6::*Nluc*, and T7::*Nluc* generated detectable positive luminescence signals only in the presence of BL21, while all co-cultures with other pathogenic strains remained negative (Fig. 6). These findings indicated that none of the reporter phages recognized or lysed the other common pathogens, exhibiting high specificity within the tested panel.

#### Detection coverage

Whole-genome sequencing analysis revealed that the 177 isolates encompassed 53 STs and 77 serotypes (Fig. S10), including the most prevalent UTI-associated STs: ST131 (*n* = 27) (40), ST1193 (*n* = 21) (41), and ST69 (*n* = 11) (42). The phylogenetic tree constructed based on whole-genome sequences of *E. coli* showed that the 177 isolates clustered into multiple distinct phylogenetic clades, with strains from the two sampling periods interspersed across the tree. These findings demonstrate the extensive genetic diversity and adequate representativeness of prevalent clinical UTIs *E. coli* lineages, supporting their suitability as an ideal strain panel for evaluating the detection coverage of individual reporter phages and their cocktail.

Following sucrose density gradient centrifugation, T2::*Nluc*, T4::*Nluc*, T5::*Nluc*, T6::*Nluc*, and T7::*Nluc* maintained high titers accompanied by significantly diminished background luminescence (Fig. S9). Upon co-culture with 177 *E. coli* strains isolated from clinical urine samples, the corresponding detection coverage for clinical strains was determined as 63.28% (112/177), 34.46% (61/177), 41.24% (73/177), 44.63% (79/177), and 36.16% (64/177), respectively (Fig. S11 to S15).

In conjunction with sequence analysis of the 177 clinical isolates, the predominant MLST and serotypes of strains detected versus missed by each reporter phage displayed favorable complementarity (Table 2). Specifically, the MLST of strains detectable by T2::*Nluc* was mainly ST1193, ST648, and ST69, and the serotypes were mainly O75:H5, O1:H6, and O102:H6. In contrast, the characteristic detectable strains of T4::*Nluc* were dominated by ST73 and ST95, with serotype O18:H5. T5::*Nluc* additionally detected the representative ST131 and ST405, accompanied by serotypes O25:H4 and O16:H5. For T6::*Nluc*, the distinct detectable MLST was ST38. Although the above reporter phages covered most of the strain types detected by T7::*Nluc*, they still recognized several unique strains, such as eco-159 (ST38, O153:H18), which was exclusively detected by T7::*Nluc* but missed by all others.

**TABLE 2:** Detection performance of reporter phages against clinical isolates^*a*^

Reporter phage	Detection coverage	Detected strains		Missed strains	
		MLST (detected/total)	Serotype (detected/total)	MLST (detected/total)	Serotype (detected/total)
T2::*Nluc*	63.28%	ST1193 (21/21) ST648 (16/17) ST69 (10/11)	O75:H5 (16/16) O1:H6 (9/9) O102:H6 (9/10)	ST131 (1/27)	O25:H4 (3/23)
T4::*Nluc*	34.46%	ST1193 (20/21) ST73 (6/6) ST95 (5/6)	O75:H5 (15/16) O18:H5 (5/5)	ST69 (0/11) ST131 (2/27) ST648 (2/17)	O25:H4 (3/23) O102:H6 (1/10) O1:H6 (1/9)
T5::*Nluc*	41.24%	ST131 (17/27) ST405 (6/9) ST95 (6/6)	O25:H4 (14/23) O102:H6 (7/10) O16:H5 (6/8)	ST648 (0/17) ST69 (1/11) ST1193 (3/21)	O1:H6 (0/9) O75:H5 (3/16)
T6::*Nluc*	44.63%	ST1193 (21/21) ST73 (6/6) ST38 (6/8)	O75:H5 (16/16) O18:H5 (5/5)	ST131 (5/27) ST648 (2/17)	O25:H4 (2/23) O1:H6 (0/9)
T7::*Nluc*	36.16%	ST38 (6/8) ST405 (6/9) ST648 (8/17)	O75:H5 (9/16) O102:H6 (6/10) O1:H6 (5/9)	ST69 (0/11) ST131 (5/27)	O25:H4 (3/23) O18:H5 (0/5) O16:H5 (3/8)

^*a*^ MLST, multi-locus sequence typing.

### Detection performance of the reporter phage cocktail

**Fig 7:**
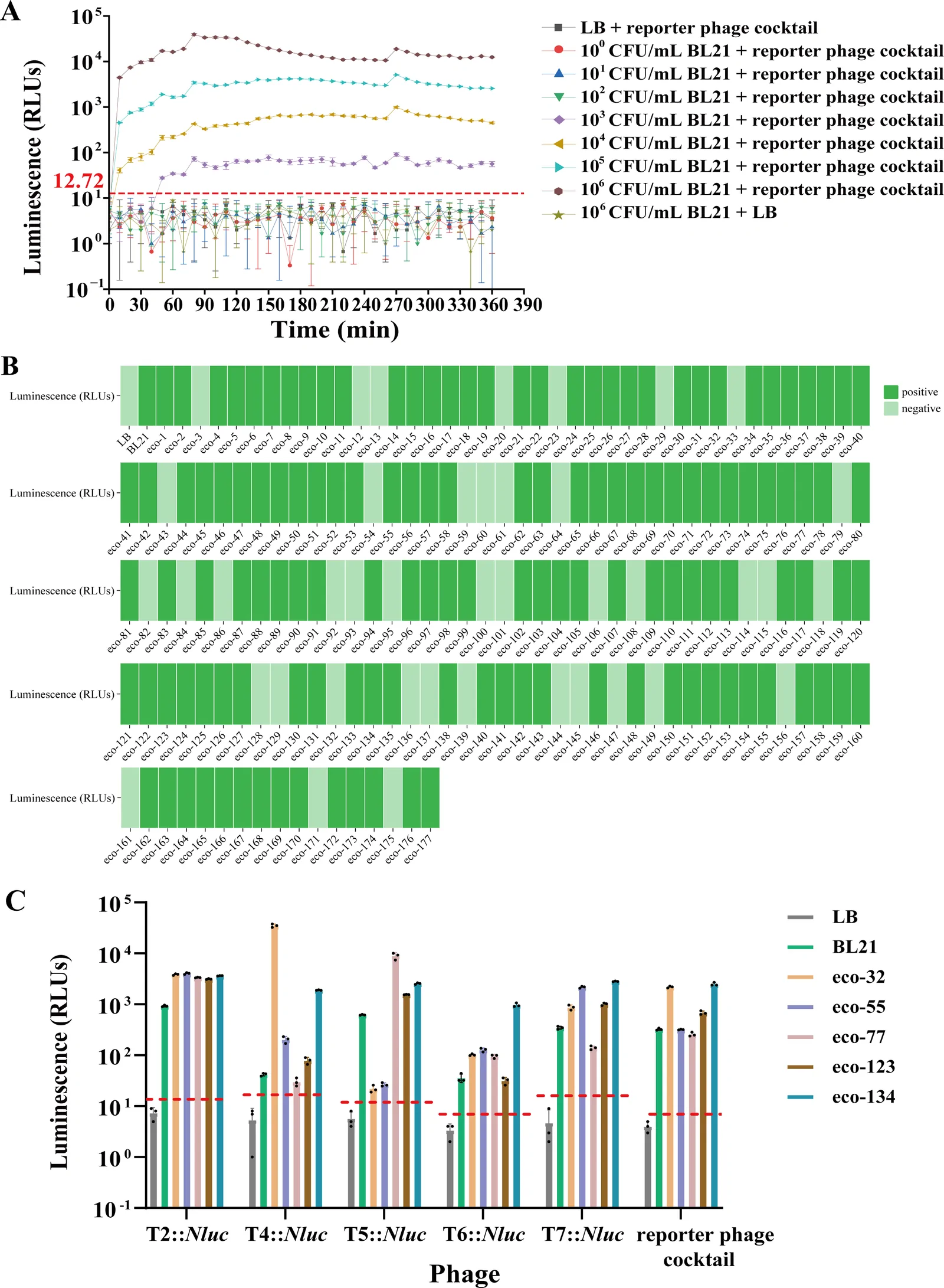
Detection performance of the reporter phage cocktail. (**A**) Limit of detection for the reporter phage cocktail. Positivity threshold = 12.72 RLU; BL21 of ≥10³ CFU/mL was detectable within 50 min. (**B**) Detection coverage of the reporter phage cocktail against clinical isolates. Positivity threshold = 10.00 RLU. (**C**) Limit of detection validation using clinical isolates. All *E. coli* samples were adjusted to a concentration of 10⁴ CFU/mL. The positivity thresholds were determined as 13.58, 16.69, 11.91, 6.80, 16.03, and 7.00 RLU for T2::*Nluc*, T4::*Nluc*, T5::*Nluc*, T6::*Nluc*, T7::*Nluc*, and the reporter phage cocktail, respectively. Positive luminescence signals were detected for all clinical strains shown in the figure.

Subsequent to co-culture of serially diluted BL21 with the reporter phage cocktail, ≥10³ CFU/mL of *E. coli* was detectable within 50 min (Fig. 7A), satisfying the diagnostic criteria for UTIs. Compared with individual reporter phages, the cocktail markedly enhanced detection coverage against 177 clinical isolates to 75.71% (134/177) (Fig. 7B) (*P* < 0.05). Among the 177 *E. coli* isolates, the reporter phage cocktail successfully detected all isolates of ST1193 (*n* = 21), ST405 (*n* = 9), ST38 (*n* = 8), and ST95 (*n* = 6), as well as all serotypes O75:H5 (*n* = 16), O102:H6 (*n* = 10), and O1:H6 (*n* = 9). However, 24.29% of the strains remained undetectable, predominantly belonging to ST131 and serotype O25:H4.

Among the 177 clinical *E. coli* isolates, eco-32, eco-55, eco-77, eco-123, and eco-134 were simultaneously recognized by T2::*Nluc*, T4::*Nluc*, T5::*Nluc*, T6::*Nluc*, T7::*Nluc,* and the reporter phage cocktail. Luminescence signals revealed that each single reporter phage, as well as the cocktail, could detect clinical *E. coli* at a concentration of 10⁴ CFU/mL after 4 h of incubation (Fig. 7C), and thus this system is applicable to clinical detection of urinary tract infection pathogens.

### Validation with clinical urine samples

In large-scale validation using clinical urine specimens, the detection sensitivities of T2::*Nluc*, T4::*Nluc*, T5::*Nluc*, T6::*Nluc*, and T7::*Nluc* for *E. coli* were 39.29%, 36.59%, 25.00%, 48.84%, and 40.00%, respectively, compared with the gold-standard methods of urine culture and MALDI-TOF MS (Tables S3 to S7). These results were primarily attributed to the restricted host ranges of individual reporter phages against clinically diverse *E. coli* clones. Notably, all assays exhibited 100% detection specificity, consistent with the high intrinsic host specificity of phages. The NPVs for *E. coli* were 93.66%, 92.22%, 92.28%, 94.71%, and 92.20%, while the PPVs were uniformly 100% for T2::*Nluc*, T4::*Nluc*, T5::*Nluc*, T6::*Nluc*, and T7::*Nluc*, respectively (Table 4). All diagnostic performance metrics are reported with corresponding 95% CIs listed in Table 4.

By combining the above reporter phages into a cocktail, the detection sensitivity reached 73.15% (Table 3), with a maintained specificity of 100%, an NPV of 96.42%, and a PPV of 100%. The overall detection performance was significantly superior to that of individual reporter phages. Furthermore, the luminescence assay based on the reporter phage cocktail achieved an accuracy of 96.74% and exhibited excellent concordance with the gold-standard method (Kappa = 0.83) (Table 4).

**TABLE 3:** Comparison of detection results between the reporter phage cocktail luminescence assay and gold-standard methods

Luminescence assay	Urine culture and MALDI-TOF MS (gold standard)		Total
	Positive^*a*^	Negative^*b*^	
Positive	79	0	79
Negative	29	782	811
Total	108	782	890

^*a*^ Positive (gold standard). Among 108 samples, 3 were polymicrobial infections containing *E. coli*.

^*b*^ Negative (gold standard). Among the 782 samples, 533 were sterile specimens, and the others were non-*E. coli* infection samples.

**TABLE 4:** Systematic profiling of detection performance of single reporter phages and their cocktail^*a*^

Parameter	T2::*Nluc*	T4::*Nluc*	T5::*Nluc*	T6::*Nluc*	T7::*Nluc*	Reporter phage cocktail
Limit of detection (CFU/mL)	≥10^1^	≥10^4^	≤10	≥10^4^	≤10	≥10^3^
Detection time (min)	220	220	310	40	300	50
Detection coverage	63.28% (112/177)	34.46% (61/177)	41.24% (73/177)	44.63% (79/177)	36.16% (64/177)	75.71% (134/177)
Sensitivity (95% CI)	39.29% (26.50%–53.25%)	36.59% (26.22%–47.95%)	25.00% (14.39%–38.37%)	48.84% (33.31%–64.54%)	40.00% (29.20%–51.56%)	73.15% (63.76%–81.22%)
Specificity (95% CI)	100.00% (99.27%–100.00%)	100.00% (99.40%–100.00%)	100.00% (99.27%–100.00%)	100.00% (99.07%–100.00%)	100.00% (99.35%–100.00%)	100.00% (99.53%–100.00%)
PPV (95% CI)	100.00% (84.56%–100.00%)	100.00% (88.43%–100.00%)	100.00% (76.84%–100.00%)	100.00% (83.89%–100.00%)	100.00% (89.11%–100.00%)	100.00% (95.44%–100.00%)
NPV (95% CI)	93.66% (92.28%–94.80%)	92.22% (90.95%–93.32%)	92.28% (91.13%–93.29%)	94.71% (93.04%–96.00%)	92.20% (90.81%–93.39%)	96.42% (95.18%–97.36%)
Accuracy (95% CI)	93.91% (91.59%–95.74%)	92.55% (90.35%–94.39%)	92.47% (89.96%–94.52%)	94.97% (92.48%–96.82%)	92.58% (90.28%–94.48%)	96.74% (95.35%–97.81%)
Cohen’s kappa (95% CI)	0.54 (0.40–0.67)	0.50 (0.39–0.62)	0.37 (0.23–0.52)	0.63 (0.49–0.77)	0.54 (0.43–0.65)	0.83 (0.77–0.89)

^*a*^ CI, confidence interval; PPV, positive predictive value; NPV, negative predictive value.

## DISCUSSION

In this study, we constructed reporter phages T2::*Nluc*, T4::*Nluc*, T5::*Nluc*, and T6::*Nluc* by the CRISPR/Cas9 system combined with homologous recombination technology. And these reporter phages were further combined with our previously constructed T7::*Nluc* to form a reporter phage cocktail for the detection of UTI-associated *E. coli*. This method exhibited moderate sensitivity with excellent specificity, required no sample pretreatment, and significantly reduced the detection time (4 h), with satisfactory concordance compared with the gold-standard method (Kappa = 0.83). These findings highlight the potential of CRISPR/Cas9-engineered reporter phages as a promising alternative for rapid detection of UTI-associated *E. coli*.

Integration of the *Nluc* reporter gene into phage genomes exerted differential effects on the biological properties of individual phages. This was mainly attributed to factors including the DNA packaging capacity of each phage capsid (43), the gene function at the engineering site (44), and the replication cycle of the phage. Nevertheless, all reporter phages preserve their core function of specific infection and lysis of host bacteria while generating luminescent signals, making them ideal tools for pathogen detection.

The LOD for all reporter phages and their cocktail met the diagnostic threshold for UTIs (≥10⁵ CFU/mL, 10⁴–10⁵ CFU/mL requires further evaluation) and markedly shortened the turnaround time (5). For instance, T6::*Nluc* detected *E. coli* (10^4^ CFU/mL) within 40 min, while the reporter phage cocktail produced a positive luminescence signal within only 10 min. This prominent advantage would greatly shorten the clinical diagnostic window, enabling prompt initiation of targeted therapy for patients in emergency and intensive care settings, thereby avoiding delays in optimal treatment. In addition, timely and accurate pathogen identification would substantially optimize the rational and efficient use of antibiotics for clinical UTI pathogens, particularly under the growing public health threat of multidrug-resistant organisms (45).

Urine is a complex biological fluid, and its composition, ionic strength, and pH can vary considerably across patients (11). Quantification of specific components in clinical urine samples typically requires purification, concentration, or other preprocessing steps to minimize matrix interference (46). However, normal urine matrix did not interfere with the detection performance of any reporter phage in our study. These results corroborate prior research from the Braun P and Meile S teams (11, 30), confirming that the presence of urine matrix does not lead to appreciable augmentation or reduction in the luminescence signal from the Nluc reporter protein.

Previous studies report that whole blood slightly delays and attenuates luminescence from *Nluc*-expressing reporter phages, yet pathogens remain reliably detectable. Importantly, no matrix interferences, such as luminescence quenching or elevated non-specific background luminescence, were observed (11, 30). But few studies characterize the effects of pyuria or highly concentrated urine on reporter phage-based detection. Among 108 *E. coli*-positive clinical samples tested with our reporter phage cocktail, 81 cases exhibited hematuria, pyuria, or both. But the detection rate remained 73.15%, largely consistent with the 75.71% observed in 177 pure bacterial cultures. This confirms that hematuria and pyuria barely impair the detection performance. Besides, only 2 of the 108 urine specimens were highly concentrated urine, and both were unambiguously identified as *E. coli*-positive. Thus, detection of urinary tract infection pathogens via reporter phage cocktail is largely unaffected by the urine matrix and eliminates complex sample pretreatment, conferring the dual advantages of simple operation and rapid detection.

In the specificity validation experiments, although only a limited set of common non-*E*. *coli* strains was tested (one per species), the clinical urine sample validation for each individual reporter phage and their cocktail was performed on a broad range of pathogenic species. For example, among the 890 clinical urine specimens included for validation of the reporter phage cocktail, 533 were culture-negative urine samples. The remaining pathogen-positive isolates comprised *E. coli* (*n* = 108), *Candida albicans* (*n* = 6), *Proteus mirabilis* (*n* = 11), *A. baumannii* (*n* = 4), *K. pneumoniae* (*n* = 43), *P. aeruginosa* (*n* = 6), *Enterobacter cloacae* (*n* = 4), *E. faecalis* (*n* = 16), *E. faecium* (*n* = 9), *S. aureus* (*n* = 2), coagulase-negative *staphylococci* (*n* = 39), *Candida glabrata* (*n* = 2), *Candida parapsilosis* (*n* = 2), *Streptococcus agalactiae* (*n* = 5), *S. epidermidis* (*n* = 5), and other uropathogens. Each species was isolated from urine samples collected from multiple patients across distinct clinical departments. The spectrum of pathogenic species recovered from clinical validation specimens for each single reporter phage was comparable to that of the cocktail. Against this panel of strains, both individual reporter phages and the cocktail achieved 100% specificity in detection. These findings confirm that this method exhibits outstanding specificity for *E. coli* detection without cross-reactivity, which is fundamentally attributed to the phages’ precise ability to recognize host bacteria at the genus, species, and even serotype levels (47). However, this inherent characteristic also introduces a core, unavoidable limitation: the host range of a single reporter phage is relatively narrow, leading to insufficient detection coverage for various heterogeneous bacterial strains isolated in clinical settings.

In this study, T2::*Nluc*, T4::*Nluc*, T5::*Nluc*, T6::*Nluc*, and T7::*Nluc* were combined into a cocktail, effectively increasing the detection coverage of clinical strains from approximately 40% to 75.71%. Five reporter phages were mixed at equal 1:1:1:1:1 ratios to guarantee adequate titers of each phage, keeping background luminescence at around 10 RLU and maximizing detection efficiency. Without incorporating additional reporter phages and under the premise of acceptable background luminescence, this formulation represents the optimal composition of the reporter phage cocktail established in this study. In addition, T2::*Nluc*, T4::*Nluc*, T5::*Nluc*, T6::*Nluc*, and T7::*Nluc* target distinct host receptors, corresponding to FadL (48), LPS/OmpC (27), FhuA (49), Tsx (50), and LPS (51), respectively. This diversity results in relatively weak competitive interference during host infection, thereby enhancing the complementarity of their host range. Furthermore, infection with any of these reporter phages generates luminescent signals in target bacteria. Therefore, competitive infection barely interferes with signal interpretation for pathogen detection.

Detection results of each reporter phage against the 177 clinical *E. coli* isolates revealed that phage-mediated detection exhibited certain lineage dependence (Table 2). The observed discrepancies across sequence types and serotypes may stem from altered availability or accessibility of phage receptors, including variations in O-antigens, capsules, outer membrane architectures, and other strain-specific defense mechanisms (52, 53). Nevertheless, substantial subclonal heterogeneity persists among isolates with identical MLSTs or serotypes (22). While individual reporter phages exhibit lineage-biased host tropism, complete identification of all its preferential isolates is unattainable. Thus, MLST and serotyping cannot accurately predict phage susceptibility independently but act as auxiliary references.

Analysis of MLST profiles and serotypes confirmed host range synergy among reporter phages and pinpointed cocktail-undetectable strains, dominated by pandemic ST131/O25:H4 isolates. The low infectivity of the reporter phages T2::*Nluc*, T4::*Nluc*, T6::*Nluc*, and T7::*Nluc* against ST131 *E. coli* accounts for this observation. This phenotype is likely attributable to reduced OmpC protein levels in ST131 strains (53), which is consistent with the findings of Gibson SB (54). The receptor of T5::*Nluc* is the FhuA protein, which is highly conserved in ST131 (O25:H4) *E. coli* and enables efficient infection (52). Nevertheless, T5::*Nluc* was diluted during the formulation of the reporter phage cocktail, coupled with substantial subclonal diversity within the ST131 *E. coli* (22), incomplete detection of ST131 strains was ultimately observed. Future optimization of the reporter phage cocktail can target the undetected strains to broaden coverage of prevalent clinical *E. coli*. One viable candidate resource is a set of hospital wastewater-isolated phages (EC.W2-1, EC.W2-6, EC.W13-3, and EC.W14-3) (55), which exhibit potent lytic activity against ST131 *E. coli*.

Beyond the cocktail strategy, Salazar KC et al. constructed a continuous cyclic co-culture bioreactor to serially passage phage *ϕ*HP3 between susceptible ST131 *E. coli* and phage-resistant ST131 variants (56), yielding the evolved phage *ϕ*HP3.1 with point mutations exclusively in the spike protein and long tail fiber genes. The elevated surface positive charge of *ϕ*HP3.1 enables it to recognize all phage-resistant mutants while retaining lytic activity against wild-type strains, effectively eliminating detection gaps for resistant ST131 isolates. In addition, multi-phage breeding mitigates false detection of ST131/O25:H4 strains. Loose M et al. bred a four-phage *Myoviridae* cocktail on diverse UPEC strains, including ST131 isolates (57), generating mosaic ε²-phages with genome-wide homologous recombination and spontaneous point mutations in tail fiber coding regions. These phages broadened their kinetic host range by up to 23% and exhibited enhanced lytic potency against phylogenetic group B2 strains, including ST131, at low MOI.

In addition, the team of Zheng X employed homologous recombination to replace the PB3 and PB4 regions of phage PH204 with the corresponding long-tail fiber from phage SP76 (58). The resulting progeny phages, RPA_1-3_ and RPB_1-3_, exhibited an expanded host range, increasing from 30 to 54 strains. Similarly, the Yehl K team utilized a site-directed mutagenesis strategy to construct a library containing millions of receptor binding protein mutants on a common T3 phage scaffold (59), from which infectious phages with expanded or altered host ranges were generated. Collectively, these studies offer innovative technical routes and experimental support for eliminating false-negative results of ST131 *E. coli*. Future research may synergistically combine co-cultivation evolution, genetic engineering, and phage cocktail strategies to improve the clinical suitability and practical efficacy of reporter phages.

In recent years, researchers worldwide have achieved continuous breakthroughs in the detection of UTI pathogens, markedly reducing detection time. Nevertheless, different technologies still face distinct practical challenges (Table 5). In our study, the detection method based on the reporter phage cocktail also achieved a significantly shortened turnaround time of approximately 4 h. Validation using large-scale clinical urine samples demonstrated that the assay reached an identification sensitivity of 73.15%, which is higher than the 68% sensitivity reported in previous similar studies (11), together with a specificity of 100%. The results showed great concordance with the gold-standard method for UTIs. Meanwhile, this method exhibits distinct advantages, including simple operation, low equipment requirements, and economical cost. Furthermore, the technique specifically detects viable *E. coli* in urine, allowing a more accurate reflection of the disease status in clinical patients. It thus holds unique value in guiding clinical treatment and dynamically monitoring therapeutic efficacy, revealing broad prospects for clinical translation and application.

**TABLE 5:** Comparison of novel detection technologies for uropathogens

Detection principle	Detection target	Detection time	Limit of detection	Advantage	Disadvantage	Reference
An isothermal amplification platform integrated phage strand exchange amplification with CRISPR/Cas12a	*Pseudomonas aeruginosa*	3.5 h	10^3^ CFU/mL	Sensitive, rapid, specific, accurate, viable-bacteria-targeting, self-amplifying signal, and professional equipment-free	Complex operational procedures, potential interference of urine components, limited host range	(60)
Electrochemical oxidation-reduction reaction based on a bare boron-doped diamond electrode	Sodium nitrite (NaNO₂), a characteristic metabolic product of pathogenic bacteria	~10 min	0.82 mg/L	Cost-effective, sensitive, rapid, simple, and low equipment requirement	Susceptible to ascorbic acid interference, lack of pathogenic bacteria identification specificity, inability to differentiate viable from non-viable bacteria	(61)
Digital β-D-glucuronidase antimicrobial susceptibility testing assay	*E. coli*	4.5 h	39 CFU/mL	Enables simultaneous specific identification, quantification, antibiotic susceptibility testing, and minimum inhibitory concentration determination of pathogenic bacteria, with viable/non-viable bacterial differentiation capability	Requirement for urine sample pretreatment involving centrifugation and bacterial isolation, challenges associated with chip production and cost control, dependence on specialized equipment	(62)
Attenuated total reflection-Fourier-transform infrared spectroscopy	Pathogen-unique cell wall/intracellular constituents	<10 min	–^*a*^	No complex sample pretreatment required, low cost, and capable of both screening and preliminary identification	Requirement for expert interpretation, susceptible to urine matrix interference, limited pathogen identification accuracy	(63)
Smartphone-assisted microfluidic paper analytical device	*E. coli*	<30 s	10 CFU/mL	Low-cost, point-of-care testing-compatible, specific, sensitive	Inconsistent results caused by smartphone performance variations, inability to differentiate viable from non-viable bacteria	(46)
Microfluidic electrochemical biosensor for performing Loop-mediated isothermal amplification	*E. coli*	1 h	24 CFU/mL	No need for immobilized probes, DNA extraction-free, rapid, sensitive	Elevated cost, high equipment requirements, potential interference of urine, inability to differentiate viable from non-viable bacteria	(64)

^*a*^ The symbol "–" indicates that only urine samples with high pathogen loads were used for qualitative discrimination in the cited study, and no specific limit of detection values were provided.

In addition to diagnosing UTIs, the reporter phage cocktail-based detection method for *E*. *coli* exhibits broad application in the timely and specific diagnosis of many other types of clinical infectious diseases, such as enteritis, sepsis, and neonatal meningitis caused by *E. coli*. Furthermore, the reporter phage constructed in this study can be combined with other pathogen-specific reporter phages, such as those targeting *S. aureus* (ISP, MP115) (65), *K. pneumoniae* (rTUN1::*nLuc*) (30), or *E. faecalis* (EfS3::*nluc*, EfS7::*nluc*) (11), to enable detection of polymicrobial mixed infections. Besides, co-culturing reporter phages or their cocktails with *E. coli* and antibiotics, the changes in luminescence signals can also reflect bacterial antibiotic susceptibility in real time, thereby facilitating the rational use and efficient development of antibiotics. For instance, Jain P identified drug-resistant *Mycobacterium tuberculosis* using phage TM4-nluc (66), and Braun P performed rapid real-time drug susceptibility testing directly on *K. pneumoniae* K64 with phage rTUN1::*nLuc* (30). With the increasing prevalence of pathogenic bacterial resistance, phages have emerged as one of the most promising alternative therapeutic strategies (67), such as in the treatment of drug-resistant *Mycobacterium abscessus* (68), *K. pneumoniae* (69), and *P. aeruginosa* (70). Notably, while exerting therapeutic effects, reporter phages can also assist in therapeutic efficacy monitoring and guidance for optimal therapeutic dose adjustment through changes in luminescence signals.

Therefore, by deeply integrating the rapid and accurate detection capabilities of reporter phages or cocktails with real-time antimicrobial susceptibility testing and targeted, efficient therapeutic functions, it is possible to address core challenges in infectious disease management, such as prolonged diagnostic time and empirical treatment. This approach can also help overcome the clinical dilemma of bacterial antimicrobial resistance and establish a comprehensive workflow encompassing sample reception-precision diagnosis-resistance analysis-targeted phage or adjunctive antibiotic therapy. Ultimately, it enables a shift from conventional pathogen isolation techniques toward an integrated diagnostic-therapeutic strategy.

## Data Availability

All data generated or analyzed during this study are included in this published article and its supplemental files.

## SUPPLEMENTAL MATERIAL

Supplemental figures spectrum.00996-26-s0001.docx Fig. S1 to S15.

Table S1 spectrum.00996-26-s0002.xlsx Summary of primers.

Table S2 spectrum.00996-26-s0003.xlsx Summary of sgRNAs.

Supplemental tables spectrum.00996-26-s0004.docx Tables S3 to S7.

ASM does not own the copyrights to Supplemental Material that may be linked to, or accessed through, an article. The authors have granted ASM a non-exclusive, world-wide license to publish the Supplemental Material files. Please contact the corresponding author directly for reuse.

## References

[1] Larcher R, Dinh A, Monnin B, Laffont-Lozes P, Loubet P, Lavigne J-P, Bruyere F, Sotto A. 2026. Phage therapy in patients with urinary tract infections: a systematic review. Expert Rev Anti Infect Ther 24:627–638. doi:10.1080/14787210.2025.2513459 40435529

[2] Yang X, Chen H, Zheng Y, Qu S, Wang H, Yi F. 2022. Disease burden and long-term trends of urinary tract infections: a worldwide report. Front Public Health 10:888205. doi:10.3389/fpubh.2022.888205 PMC9363895 35968451

[3] Bellankimath AB, Branders S, Kegel I, Ali J, Asadi F, Johansen TEB, Imirzalioglu C, Hain T, Wagenlehner F, Ahmad R. 2025. Metagenomic sequencing enables accurate pathogen and antimicrobial susceptibility profiling in complicated UTIs in approximately four hours. Nat Commun 17:187. doi:10.1038/s41467-025-66865-8 41339341 PMC12780005

[4] Chan CCY, Gregson DB, Wildman SD, Bihan DG, Groves RA, Aburashed R, Rydzak T, Pittman K, Van Bavel N, Lewis IA. 2025. Metabolomics strategy for diagnosing urinary tract infections. Nat Commun 16:2658. doi:10.1038/s41467-025-57765-y 40102424 PMC11920235

[5] Moreland RB, Brubaker L, Tinawi L, Wolfe AJ. 2025. Rapid and accurate testing for urinary tract infection: new clothes for the emperor. Clin Microbiol Rev 38:e0012924. doi:10.1128/cmr.00129-24 40366169 PMC12160498

[6] Kurt H, Soylukan C, Çelik S, Çapkın E, Acuner IC, Topkaya AE, Yüce M. 2025. Rapid and sensitive biosensing of uropathogenic *E. coli* using plasmonic nanohole arrays on MIM: bridging the gap between lab and clinical diagnostics. Biosens Bioelectron 280:117419. doi:10.1016/j.bios.2025.117419 40174438

[7] Yeh F-W, Chiu C-H, Wang R, Su Y-C, Virly, Lin T-Y. 2025. Development of bacteriophage-modified europium alginate beads for rapid screening of *Escherichia coli*. Int J Biol Macromol 302:140415. doi:10.1016/j.ijbiomac.2025.140415 39890000

[8] Szlachta-McGinn A, Douglass KM, Chung UYR, Jackson NJ, Nickel JC, Ackerman AL. 2022. Molecular diagnostic methods versus conventional urine culture for diagnosis and treatment of urinary tract infection: a systematic review and meta-analysis. Eur Urol Open Sci 44:113–124. doi:10.1016/j.euros.2022.08.009 36093322 PMC9459428

[9] Alonzo LF, Hinkley TC, Miller A, Calderon R, Garing S, Williford J, Clute-Reinig N, Spencer E, Friend M, Madan D, Dinh VTT, Bell D, Weigl BH, Nugen SR, Nichols KP, Le Ny A-LM. 2022. A microfluidic device and instrument prototypes for the detection of *Escherichia coli* in water samples using a phage-based bioluminescence assay. Lab Chip 22:2155–2164. doi:10.1039/d1lc00888a 35521688

[10] Moon K, Coxon C, Årdal C, Botgros R, Djebara S, Durno L, Fiore CR, Perrin J-B, Dixon DM, Cavaleri M. 2025. Considerations and perspectives on phage therapy from the transatlantic taskforce on antimicrobial resistance. Nat Commun 16:10883. doi:10.1038/s41467-025-64608-3 41345089 PMC12678583

[11] Meile S, Du J, Staubli S, Grossmann S, Koliwer-Brandl H, Piffaretti P, Leitner L, Matter CI, Baggenstos J, Hunold L, Milek S, Guebeli C, Kozomara-Hocke M, Neumeier V, Botteon A, Klumpp J, Marschall J, McCallin S, Zbinden R, Kessler TM, Loessner MJ, Dunne M, Kilcher S. 2023. Engineered reporter phages for detection of *Escherichia coli*, *Enterococcus*, and *Klebsiella* in urine. Nat Commun 14:4336. doi:10.1038/s41467-023-39863-x 37474554 PMC10359277

[12] Santos SB, Costa MJ, Meneses L, Pires DP. 2025. Improved *Pseudomonas aeruginosa* detection through the development of an engineered reporter bacteriophage. Biosens Bioelectron 289:117907. doi:10.1016/j.bios.2025.117907 40889470

[13] Gong Q, Song B, Zhao R, Ma R, Wang D, Qin X, Li Y, Chen W, Ma L, Menghwar H, Huang C, Ma Q. 2025. Bioluminescent-reporter phagosensor for quantitative detection of viable *Escherichia coli* in water. Water Res 285:124151. doi:10.1016/j.watres.2025.124151 40633416

[14] Li M, Hao Z, Yan J, Chen X, Li H, Wang C, Wang C. 2025. Rapid detection of *Escherichia coli* in bloodstream infection via CRISPR-Cas9 engineered reporter phage T7:: Nluc and microfluidic chip platform. Acta Biochim Biophys Sin (Shanghai) 58:792–805. doi:10.3724/abbs.2025150 40999904 PMC13107020

[15] Hussain W, Ullah MW, Farooq U, Aziz A, Wang S. 2021. Bacteriophage-based advanced bacterial detection: concept, mechanisms, and applications. Biosens Bioelectron 177:112973. doi:10.1016/j.bios.2021.112973 33429203

[16] García-García JD, Contreras-Alvarado LM, Cruz-Córdova A, Hernández-Castro R, Flores-Encarnacion M, Rivera-Gutiérrez S, Arellano-Galindo J, A Ochoa S, Xicohtencatl-Cortes J. 2025. Pathogenesis and immunomodulation of urinary tract infections caused by uropathogenic *Escherichia coli*. Microorganisms 13:745. doi:10.3390/microorganisms13040745 40284582 PMC12029274

[17] Carter C, Hutchison A, Rudder S, Trotter E, Waters EV, Elumogo N, Langridge GC. 2023. Uropathogenic *Escherichia coli* population structure and antimicrobial susceptibility in Norfolk, UK. J Antimicrob Chemother 78:2028–2036. doi:10.1093/jac/dkad201 37358190 PMC10393884

[18] Alghoribi MF, Gibreel TM, Farnham G, Al Johani SM, Balkhy HH, Upton M. 2015. Antibiotic-resistant ST38, ST131 and ST405 strains are the leading uropathogenic *Escherichia coli* clones in Riyadh, Saudi Arabia. J Antimicrob Chemother 70:2757–2762. doi:10.1093/jac/dkv188 26183183

[19] Phan M-D, Schirra HJ, Nhu NTK, Peters KM, Sarkar S, Allsopp LP, Achard MES, Kappler U, Schembri MA. 2024. Combined functional genomic and metabolomic approaches identify new genes required for growth in human urine by multidrug-resistant *Escherichia coli* ST131. mBio 15:e0338823. doi:10.1128/mbio.03388-23 38353545 PMC10936160

[20] Pitout JDD, Peirano G, Chen L, DeVinney R, Matsumura Y. 2022. *Escherichia coli* ST1193: following in the footsteps of *E. coli* ST131. Antimicrob Agents Chemother 66:e0051122. doi:10.1128/aac.00511-22 35658504 PMC9295538

[21] Bighi NM, Fonseca ÉL, Freitas FS, Morgado SM, Vicente ACP. 2025. Pandemic ST131 *Escherichia coli* presenting the UPEC/EAEC and ExPEC/EAEC hybrid pathotypes recovered from extraintestinal infections in a clinical setting of the Brazilian Amazon region. Mem Inst Oswaldo Cruz 120:e240204. doi:10.1590/0074-02760240204 PMC11984961 40243867

[22] Nicolas-Chanoine M-H, Bertrand X, Madec J-Y. 2014. *Escherichia coli* ST131, an intriguing clonal group. Clin Microbiol Rev 27:543–574. doi:10.1128/CMR.00125-13 24982321 PMC4135899

[23] Chen H, Liu H, Gong Y, Dunstan RA, Ma Z, Zhou C, Zhao D, Tang M, Lithgow T, Zhou T. 2024. A *Klebsiella*-phage cocktail to broaden the host range and delay bacteriophage resistance both *in vitro* and *in vivo*. NPJ Biofilms Microbiomes 10:127. doi:10.1038/s41522-024-00603-8 39543151 PMC11564825

[24] Chen Y, Wang X, Zhang Q, Zhang Z, Liu Z, Zhao X, Li L, Hu M, Lv Q, Luo Y, Xu X, Petersen B, Cai Y, Sicheritz-Pontén T, Clokie MRJ, Liu Y. 2025. ABCD-type phage cocktail targeting distinct LPS receptor sites demonstrates superior efficacy against multidrug-resistant *Salmonella*. J Hazard Mater 500:140435. doi:10.1016/j.jhazmat.2025.140435 41240830

[25] Zhu H, Rahman SU, Pan T, Yuan J, Zhou X. 2025. Dark-field intelligent detection of *V. parahaemolyticus* using T4 bacteriophage displaying tail spike proteins and gold nanoparticles. Biosens Bioelectron 288:117830. doi:10.1016/j.bios.2025.117830 40749397

[26] Tanji Y, Murofushi K, Miyanaga K. 2005. Quick selection of a chimeric T2 phage that displays active enzyme on the viral capsid. Biotechnol Prog 21:1768–1771. doi:10.1021/bp050201c 16321064

[27] Subedi D, Barr JJ. 2021. Temporal stability and genetic diversity of 48-year-old T-series phages. mSystems 6:e00990-20. doi:10.1128/mSystems.00990-20 33594003 PMC8573959

[28] Ramirez-Chamorro L, Boulanger P, Rossier O. 2021. Strategies for bacteriophage T5 mutagenesis: expanding the toolbox for phage genome engineering. Front Microbiol 12:667332. doi:10.3389/fmicb.2021.667332 33981295 PMC8108384

[29] Duong MM, Carmody CM, Ma Q, Peters JE, Nugen SR. 2020. Optimization of T4 phage engineering via CRISPR/Cas9. Sci Rep 10:18229. doi:10.1038/s41598-020-75426-6 33106580 PMC7588440

[30] Braun P, Raab R, Bugert JJ, Braun S. 2023. Recombinant reporter phage rTUN1::*nLuc* enables rapid detection and real-time antibiotic susceptibility testing of *Klebsiella pneumoniae* K64 strains. ACS Sens 8:630–639. doi:10.1021/acssensors.2c01822 36719711 PMC9972469

[31] Meile S, Kilcher S, Loessner MJ, Dunne M. 2020. Reporter phage-based detection of bacterial pathogens: design guidelines and recent developments. Viruses 12:944. doi:10.3390/v12090944 32858938 PMC7552063

[32] Gibb B, Hyman P, Schneider CL. 2021. The many applications of engineered bacteriophages—an overview. Pharmaceuticals (Basel) 14:634. doi:10.3390/ph14070634 34208847 PMC8308837

[33] Vu TN, Clark JR, Jang E, D’Souza R, Nguyen LP, Pinto NA, Yoo S, Abadie R, Maresso AW, Yong D. 2024. Appelmans protocol - A directed *in vitro* evolution enables induction and recombination of prophages with expanded host range. Virus Res 339:199272. doi:10.1016/j.virusres.2023.199272 37981215 PMC10730860

[34] Hao X, Cen X, He M, Wen Y, Zhang H. 2023. Isolation, biological and whole genome characteristics of a *Proteus mirabilis* bacteriophage strain. BMC Microbiol 23:215. doi:10.1186/s12866-023-02960-4 PMC10410936 37553593

[35] Dong Z, Wu R, Liu L, Ai S, Yang J, Li Q, Fu K, Zhou Y, Fu H, Zhou Z, Liu H, Zhong Z, Qiu X, Peng G. 2024. Phage P2-71 against multi-drug resistant *Proteus mirabilis*: isolation, characterization, and non-antibiotic antimicrobial potential. Front Cell Infect Microbiol 14:1347173. doi:10.3389/fcimb.2024.1347173 38500503 PMC10945010

[36] Koncz M, Stirling T, Hadj Mehdi H, Méhi O, Eszenyi B, Asbóth A, Apjok G, Tóth Á, Orosz L, Vásárhelyi BM, et al.. 2024. Genomic surveillance as a scalable framework for precision phage therapy against antibiotic-resistant pathogens. Cell 187:5901–5918. doi:10.1016/j.cell.2024.09.009 39332413

[37] Chang RYK, Nang SC, Chan H-K, Li J. 2022. Novel antimicrobial agents for combating antibiotic-resistant bacteria. Adv Drug Deliv Rev 187:114378. doi:10.1016/j.addr.2022.114378 35671882

[38] Taati Moghadam M, Mohebi S, Sheikhi R, Hasannejad-Bibalan M, Shahbazi S, Nemati S. 2025. Phage and endolysin therapy against antibiotics resistant bacteria: from bench to bedside. MedComm (2020) 6:e70280. doi:10.1002/mco2.70280 40661138 PMC12256688

[39] Jiang S, Li H, Zhang L, Mu W, Zhang Y, Chen T, Wu J, Tang H, Zheng S, Liu Y, Wu Y, Luo X, Xie Y, Ren J. 2025. Generic diagramming platform (GDP): a comprehensive database of high-quality biomedical graphics. Nucleic Acids Res 53:D1670–D1676. doi:10.1093/nar/gkae973 39470721 PMC11701665

[40] Halaji M, Shahidi S, Ataei B, Atapour A, Feizi A, Havaei SA. 2021. Molecular epidemiology of *bla*_CTX-M_ gene-producing uropathogenic *Escherichia coli* among Iranian kidney transplant patients: clonal dissemination of CC131 and CC10. Ann Clin Microbiol Antimicrob 20:65. doi:10.1186/s12941-021-00470-7 34496873 PMC8424993

[41] Aljohani RH, ElFeky DS, Alswaji AA, Alrashidi E, Okdah L, Alalwan B, Aljohani SM, Balkhy HH, Redhwan A, Alghoribi MF. 2023. Genomic characterization of uropathogenic *Escherichia coli* isolates from tertiary hospitals in Riyadh, Saudi Arabia. Int J Mol Sci 24:7582. doi:10.3390/ijms24087582 37108743 PMC10141978

[42] Ha J-H, Shin J-I, Kim K-M, Choi J-G, Trinh MP, Anh WJ, Kang K-M, Kang H-L, Byun J-H, Boonyanugomol W, Kwon KW, Jung MH, Baik SC, Lee W-K, Shin M-K. 2025. Prevalence and virulence profiles of ESBL-producing *Escherichia coli* in urinary and blood infections in South Korea. Folia Microbiol (Praha) 70:589–600. doi:10.1007/s12223-024-01205-9 39433645

[43] Nurmemmedov E, Castelnovo M, Medina E, Catalano CE, Evilevitch A. 2012. Challenging packaging limits and infectivity of phage λ. J Mol Biol 415:263–273. doi:10.1016/j.jmb.2011.11.015 22108169

[44] Zelcbuch L, Yitzhaki E, Nissan O, Gidron E, Buchshtab N, Kario E, Kredo-Russo S, Zak NB, Bassan M. 2021. Luminescent phage-based detection of *Klebsiella pneumoniae*: from engineering to diagnostics. Pharmaceuticals (Basel) 14:347. doi:10.3390/ph14040347 33918942 PMC8069110

[45] Abbott IJ, Peel TN, Cairns KA, Stewardson AJ. 2023. Antibiotic management of urinary tract infections in the post-antibiotic era: a narrative review highlighting diagnostic and antimicrobial stewardship. Clin Microbiol Infect 29:1254–1266. doi:10.1016/j.cmi.2022.05.016 35640839

[46] Cho S, Park TS, Nahapetian TG, Yoon J-Y. 2015. Smartphone-based, sensitive µPAD detection of urinary tract infection and gonorrhea. Biosens Bioelectron 74:601–611. doi:10.1016/j.bios.2015.07.014 26190472

[47] Meile S, Sarbach A, Du J, Schuppler M, Saez C, Loessner MJ, Kilcher S. 2020. Engineered reporter phages for rapid bioluminescence-based detection and differentiation of viable *Listeria* cells. Appl Environ Microbiol 86:e00442-20. doi:10.1128/AEM.00442-20 32245761 PMC7237785

[48] Trojet SN, Caumont-Sarcos A, Perrody E, Comeau AM, Krisch HM. 2011. The gp38 adhesins of the T4 superfamily: a complex modular determinant of the phage’s host specificity. Genome Biol Evol 3:674–686. doi:10.1093/gbe/evr059 21746838 PMC3157838

[49] Linares R, Arnaud C-A, Effantin G, Darnault C, Epalle NH, Boeri Erba E, Schoehn G, Breyton C. 2023. Structural basis of bacteriophage T5 infection trigger and *E. coli* cell wall perforation. Sci Adv 9:eade9674. doi:10.1126/sciadv.ade9674 36961893 PMC10038345

[50] Kortright KE, Doss-Gollin S, Chan BK, Turner PE. 2021. Evolution of bacterial cross-resistance to lytic phages and albicidin antibiotic. Front Microbiol 12:658374. doi:10.3389/fmicb.2021.658374 34220747 PMC8245764

[51] Garcia-Doval C, van Raaij MJ. 2012. Structure of the receptor-binding carboxy-terminal domain of bacteriophage T7 tail fibers. Proc Natl Acad Sci USA 109:9390–9395. doi:10.1073/pnas.1119719109 22645347 PMC3386108

[52] Wang Y, Chen X, Hu Y, Zhu G, White AP, Köster W. 2018. Evolution and sequence diversity of FhuA in *Salmonella* and *Escherichia*. Infect Immun 86:e00573-18. doi:10.1128/IAI.00573-18 PMC6204707 30150258

[53] Suelter CS, Hanson ND. 2020. OmpC regulation differs between ST131 and non-ST131 *Escherichia coli* clinical isolates and involves differential expression of the small RNA MicC. J Antimicrob Chemother 75:1151–1158. doi:10.1093/jac/dkz566 31998951 PMC7177473

[54] Gibson SB, Green SI, Liu CG, Salazar KC, Clark JR, Terwilliger AL, Kaplan HB, Maresso AW, Trautner BW, Ramig RF. 2019. Constructing and characterizing bacteriophage libraries for phage therapy of human infections. Front Microbiol 10:2537. doi:10.3389/fmicb.2019.02537 31781060 PMC6861333

[55] Shamsuzzaman M, Kim S, Kim J. 2024. Bacteriophage as a novel therapeutic approach for killing multidrug-resistant *Escherichia coli* ST131 clone. Front Microbiol 15:1455710. doi:10.3389/fmicb.2024.1455710 39726968 PMC11670814

[56] Salazar KC, Ma L, Green SI, Zulk JJ, Trautner BW, Ramig RF, Clark JR, Terwilliger AL, Maresso AW. 2021. Antiviral resistance and phage counter adaptation to antibiotic-resistant extraintestinal pathogenic *Escherichia coli*. mBio 12:e00211-21. doi:10.1128/mBio.00211-21 33906920 PMC8092219

[57] Loose M, Sáez Moreno D, Mutti M, Hitzenhammer E, Visram Z, Dippel D, Schertler S, Tišáková LP, Wittmann J, Corsini L, Wagenlehner F. 2021. Natural bred ε^2^-phages have an improved host range and virulence against uropathogenic *Escherichia coli* over their ancestor phages. Antibiotics (Basel) 10:1337. doi:10.3390/antibiotics10111337 34827275 PMC8614997

[58] Zheng X, Wang X, Li P, Zhou Y, Zhu X, Hu Z, Wang H, Chen M, Huo X, Liu Y, Zhang W. 2025. The change of long tail fibers expanded the host range of a T5-like *Salmonella* phage and its application in milk. BMC Microbiol 25:169. doi:10.1186/s12866-025-03895-8 PMC11938639 40133802

[59] Yehl K, Lemire S, Yang AC, Ando H, Mimee M, Torres MDT, de la Fuente-Nunez C, Lu TK. 2019. Engineering phage host-range and suppressing bacterial resistance through phage tail fiber mutagenesis. Cell 179:459–469. doi:10.1016/j.cell.2019.09.015 31585083 PMC6924272

[60] Xiao X, Zhang C, Zhang L, Zuo C, Wu W, Cheng F, Wu D, Xie G, Mao X, Yang Y. 2025. A phage amplification-assisted SEA-CRISPR/Cas12a system for viable bacteria detection. J Mater Chem B 13:1372–1382. doi:10.1039/D4TB02178A 39663988

[61] Zhang Z, Ogata G, Asai K, Yamamoto T, Einaga Y. 2023. Electrochemical diagnosis of urinary tract infection using boron-doped diamond electrodes. ACS Sens 8:4245–4252. doi:10.1021/acssensors.3c01569 37880948

[62] Wu W, Zhao Q, Cai G, Zhang B, Suo Y, Liu Y, Jin W, Mu Y. 2022. All-in-one *Escherichia coli* viability assay for multi-dimensional detection of uncomplicated urinary tract infections. Anal Chem 94:17853–17860. doi:10.1021/acs.analchem.2c03604 36524619

[63] Pan S-W, Lu H-C, Lo J-I, Ho L-I, Tseng T-R, Ho M-L, Cheng B-M. 2022. Using an ATR-FTIR technique to detect pathogens in patients with urinary tract infections: a pilot study. Sensors 22:3638. doi:10.3390/s22103638 35632048 PMC9147530

[64] Safavieh M, Ahmed MU, Tolba M, Zourob M. 2012. Microfluidic electrochemical assay for rapid detection and quantification of *Escherichia coli*. Biosens Bioelectron 31:523–528. doi:10.1016/j.bios.2011.11.032 22177893

[65] Brown M, Hahn W, Bailey B, Hall A, Rodriguez G, Zahn H, Eisenberg M, Erickson S. 2020. Development and evaluation of a sensitive bacteriophage-based MRSA diagnostic screen. Viruses 12:631. doi:10.3390/v12060631 32545159 PMC7354448

[66] Jain P, Garing S, Verma D, Saranathan R, Clute-Reinig N, Gadwa J, Peterson C, Hermansky G, Astashkina Fernandez A, Asare E, Weisbrod TR, Spencer E, Mulholland CV, Berney M, Bell D, Nichols KP, Le Ny A-LM, Ordway D, Jacobs WR Jr, Somoskovi A, Minch KJ. 2020. Nanoluciferase reporter mycobacteriophage for sensitive and rapid detection of *Mycobacterium tuberculosis* drug susceptibility. J Bacteriol 202:e00411-20. doi:10.1128/JB.00411-20 32900827 PMC7585058

[67] Strathdee SA, Hatfull GF, Mutalik VK, Schooley RT. 2023. Phage therapy: from biological mechanisms to future directions. Cell 186:17–31. doi:10.1016/j.cell.2022.11.017 36608652 PMC9827498

[68] Dedrick RM, Guerrero-Bustamante CA, Garlena RA, Russell DA, Ford K, Harris K, Gilmour KC, Soothill J, Jacobs-Sera D, Schooley RT, Hatfull GF, Spencer H. 2019. Engineered bacteriophages for treatment of a patient with a disseminated drug-resistant *Mycobacterium abscessus*. Nat Med 25:730–733. doi:10.1038/s41591-019-0437-z 31068712 PMC6557439

[69] Peng X, Chang J, Zhang H, Li X, Zhang C, Jiao S, Lv C, Wang N, Zhao J, Wang B, Zhang W, Zhang Z. 2025. Isolation, characterization, and genomic analysis of a novel bacteriophage vB_Kp_XP4 targeting hypervirulent and multidrug-resistant *Klebsiella pneumoniae*. Front Microbiol 16:1491961. doi:10.3389/fmicb.2025.1491961 PMC11925924 40124894

[70] Chung SJ, Liu Y, Thong S, Zhong Y, Chong ZS, Lim Z, Tan S, Yeo JH, Koh MG, Chua NGS, et al.. 2026. Timely bespoke phage-antibiotic combination to treat refractory *Pseudomonas aeruginosa* mediastinitis and vascular graft infection. Nat Commun 17:1385. doi:10.1038/s41467-025-68136-y PMC12877064 41513696

