# Supplementary material for: Development and clinical validation of a CRISPR/Cas9-engineered reporter phage cocktail for rapid detection of Escherichia coli in urine
Source: Microbiol Spectr. 2026 Aug 6;14(9):e00996-26. doi: 10.1128/spectrum.00996-26 (PMC13532311; doi:10.1128/spectrum.00996-26)
Supplement: Supplementary file 1 [file spectrum.00996-26-s0001.docx]

**Supplementary Fig. 1 PCR amplification of the homology arms and *Nluc* gene block.**

A. PCR amplification of T2 phage homology arms and *Nluc* gene block. Lane 1: upstream homology arm, T2-UHA-F/R, 1078 bp; Lane 2: *Nluc* gene block, T2-Nluc-F/R, 671 bp; Lane 3: downstream homology arm, T2-DHA-F/R, 1063 bp. **B:** PCR amplification of T4 phage homology arms and *Nluc* gene block. Lanes 1-2: downstream homology arm, T4-DHA-F/R, 1027 bp; Lanes 3-4: *Nluc* gene block, T4-Nluc-F/R, 709 bp; Lanes 5-6: upstream homology arm, T4-UHA-F/R, 1034 bp. **C:** PCR amplification of T5 phage homology arms and *Nluc* gene block. Lane 1: upstream homology arm, T5-UHA-F/R, 948 bp; Lane 2: *Nluc* gene block, T5-Nluc-F/R, 671 bp; Lane 3: downstream homology arm, T5-DHA-F/R, 1244 bp. **D:** PCR amplification of T6 phage homology arms and *Nluc* gene block. Lane 1: downstream homology arm, T6-DHA-F/R, 1046 bp;

Lane 2: *Nluc* gene block, T6-Nluc-F/R, 669 bp; Lane 3: upstream homology arm, T6-UHA-F/R, 961 bp. M, Marker.

**Supplementary Fig. 2 Construction and validation of**

**homologous recombination plasmids.**

**A:** PCR amplification of linearized vector (T7 promoter-deleted) for T2 phage homologous recombination. T2-Vector-F/R, 3907 bp. **B:** Verification of homologous recombination plasmid for T2. Lane 1: T2-UHA-F/M13-R, 2851 bp; Lane 2: T2-UHA-F/T2-DHA-R, 2772 bp. **C:** Verification of homologous recombination plasmid for T4. Lane 1: T4-DHA-F/M13-R, 1106 bp; Lane 2: M13-F/T4-Nluc-R, 1801 bp; Lane 3: M13-F/T4-DHA-R, 2808 bp; Lane 4: T4-Nluc-F/M13-R, 1788 bp; Lane 5: M13-F/T4-UHA-R, 1119 bp; Lane 6: T4-UHA-F/M13-R, 2840 bp; Lane 7: T4-UHA-F/T4-DHA-R, 2761 bp. **D:** Verification of homologous recombination plasmid for T5. Lane 1: T5-UHA-F/M13-R, 2902 bp; Lane 2: T5-UHA-F/T5-DHA-R, 2823 bp. **E:**

Verification of homologous recombination plasmid for T6. Lane 1: M13-F/T6-DHR-R, 2683 bp; Lane 2: M13-F/T6-Nluc-R, 1657 bp; Lane 3: T6-UHR-F/T6-DHR-R, 2636 bp; Lane 4: T6-UHR-F/M13-R, 2715 bp; Lane 5: T6-Nluc-F/M13-R, 1774 bp. M, Marker.

**Supplementary Fig. 3 Construction of the CRISPR/Cas9 system.**

**A:** Verification of pCRISPR-soc-sgRNA-1~10 plasmids for T2. Lanes 1-10: T2-soc-sgRNA-1~10-F/pCRISPR-R, 646 bp. **B:** Verification of the pCRISPR-soc-cr6 plasmid for T4. T4-soc-cr6-F/pCRISPR-R, 646 bp. **C:** Verification of pCRISPR-dmp-sgRNA-1~10 plasmids for T5. Lanes 1-10: T5-dmp-sgRNA-1~10-F/pCRISPR-R, 646 bp. **D:** Verification of pCRISPR-hoc-sgRNA-1~10 plasmids for T6. Lanes 1-10: T6-hoc-sgRNA-1~10-F/pCRISPR-R, 646 bp. **E:** Verification of pCas9-BL21. pCas9-F/R, 1335 bp. M, Marker.

**Supplementary Fig. 4 Verification of reporter phages.**

**A:** Validation of the T2::*Nluc* reporter phage. Lanes 1-3: template from T2::*Nluc*, T2 recombinant primer F/R, 3123 bp; Lane 4: template from T2, T2 recombinant primer F/R, 2711 bp. **B:** Validation of the T4::*Nluc* reporter phage. Lanes 1-3: template from T4::*Nluc*, T4 recombinant primer F/R, 2837 bp; Lanes 4-5: template from T4, T4 recombinant primer F/R, 2358 bp. **C:** Validation of the T5::*Nluc* reporter phage. Lanes 1-2: template from T5::*Nluc*, T5 recombinant primer F/R, 3435 bp; Lane 3: template from T5, T5 recombinant primer F/R, 3412 bp. **D:** Validation of the T6::*Nluc* reporter phage. Lane 1: template from T6, T6 recombinant primer F/R, 3863 bp; Lanes 2-3: template from T6::*Nluc*, T6 recombinant primer F/R, 3907 bp. M: marker.

**Supplementary Fig. 5 Comparison of biological characteristics**

**between T2 and T2::*Nluc.***

**A:** One-step growth curves. RM-ANOVA, *P* < 0.05. **B:** Optimal multiplicity of infection. **C:** Lysis efficiency. Kruskal–Wallis H test, pairwise comparison between the “BL21 + T2” group and “BL21 + T2::*Nluc*” group, *P* > 0.05.

**Supplementary Fig. 6 Comparison of biological characteristics**

**between T4 and T4::*Nluc.***

**A:** One-step growth curves. RM-ANOVA, *P* > 0.05. **B:** Optimal multiplicity of infection. **C:** Lysis efficiency. Kruskal–Wallis H test, pairwise comparison between the “BL21 + T4” group and “BL21 + T4::*Nluc*” group, *P* < 0.05.

**Supplementary Fig. 7 Comparison of biological characteristics**

**between T5 and T5::*Nluc.***

**A:** One-step growth curves. RM-ANOVA, *P* < 0.05. **B:** Optimal multiplicity of infection. **C:** Lysis efficiency. Kruskal–Wallis H test, pairwise comparison between the “BL21 + T5” group and “BL21 + T5::*Nluc*” group, *P* < 0.05.

**Supplementary Fig. 8 Comparison of biological characteristics**

**between T6 and T6::*Nluc.***

**A:** One-step growth curves. RM-ANOVA, *P* < 0.05. **B:** Optimal multiplicity of infection. **C:** Lysis efficiency. Kruskal–Wallis H test, pairwise comparison between the “BL21 + T6” group and “BL21 + T6::*Nluc*” group, *P* > 0.05.

**Supplementary Fig. 9 Comparison of background luminescence signals**

**before and after sucrose density gradient centrifugation.**

**A-E:** Comparison of background luminescence signals of T2::*Nluc*, T4::*Nluc*, T5::*Nluc*, T6::*Nluc*, and T7::*Nluc* before and after sucrose density gradient centrifugation. Multiple t-tests, *****P* < 0.0001.

**Supplementary Fig. 10 Phylogenetic tree of 177 clinical urinary *E. coli* isolates.**

Isolates with multiple O-antigen serotypes indicate all predicted O serotypes by Serotype Finder based on sequence data, which could not be assigned to a single type. Isolates labeled with only O or only H serotypes represent unresolvable predictions due to genetic variation. MLST, multi-locus sequence typing.

**Supplementary Fig. 11 Detection coverage of T2::*Nluc* against clinical isolates.** Positivity threshold = 9.00 RLU.

**Supplementary Fig. 12 Detection coverage of T4::*Nluc* against clinical isolates.** Positivity threshold = 11.92 RLU.

**Supplementary Fig. 13 Detection coverage of T5::*Nluc* against clinical isolates.** Positivity threshold = 12.95 RLU.

**Supplementary Fig. 14 Detection coverage of T6::*Nluc* against clinical isolates.** Positivity threshold = 15.55 RLU.

**Supplementary Fig. 15 Detection coverage of T7::*Nluc* against clinical isolates.** Positivity threshold = 11.25 RLU.
