# Supplementary material for: Development and clinical validation of a CRISPR/Cas9-engineered reporter phage cocktail for rapid detection of Escherichia coli in urine
Source: Microbiol Spectr. 2026 Aug 6;14(9):e00996-26. doi: 10.1128/spectrum.00996-26 (PMC13532311; doi:10.1128/spectrum.00996-26)
Supplement: Supplementary file 2 [file spectrum.00996-26-s0004.docx]

**Supplementary Table 3 Comparison of detection results between T2::*Nluc***

**luminescence assay and gold-standard methods.**

| **Luminescence assay** | **Urine culture and MALDI‑TOF MS (gold-standard)** | | **total** |
| --- | --- | --- | --- |
|  | **positive^a^** | **negative^b^** |  |
| **positive** | 22 | 0 | 22 |
| **negative** | 34 | 502 | 536 |
| **total** | 56 | 502 | 558 |

^a^Positive (gold-standard). All 56 samples were confirmed as *E. coli* infections.

^b^Negative (gold-standard). Among 502 samples, 356 were sterile and the rest were non-*E. coli* infected samples.

**Supplementary Table 4 Comparison of detection results between T4::*Nluc***

**luminescence assay and gold-standard methods.**

| **Luminescence assay** | **Urine culture and MALDI‑TOF MS (gold-standard)** | | **total** |
| --- | --- | --- | --- |
|  | **positive^a^** | **negative^b^** |  |
| **positive** | 30 | 0 | 30 |
| **negative** | 52 | 616 | 668 |
| **total** | 82 | 616 | 698 |

^a^Positive (gold-standard). Among the 82 samples, 6 were mixed infections with *E. coli*.

^b^Negative (gold-standard). Among the 616 samples, 408 were sterile, and the remainder were non-*E. coli* infection samples.

**Supplementary Table 5 Comparison of detection results between T5::*Nluc***

**luminescence assay and gold-standard methods.**

| **Luminescence assay** | **Urine culture and MALDI‑TOF MS (gold-standard)** | | **total** |
| --- | --- | --- | --- |
|  | **positive^a^** | **negative^b^** |  |
| **positive** | 14 | 0 | 14 |
| **negative** | 42 | 502 | 544 |
| **total** | 56 | 502 | 558 |

^a^Positive (gold-standard). All 56 samples were confirmed as *E. coli* infections.

^b^Negative (gold-standard). Among 502 samples, 356 were sterile and the rest were non-*E. coli* infected samples.

**Supplementary Table 6 Comparison of detection results between T6::*Nluc***

**luminescence assay and gold-standard methods.**

| **Luminescence assay** | **Urine culture and MALDI‑TOF MS (gold-standard)** | | **total** |
| --- | --- | --- | --- |
|  | **positive^a^** | **negative^b^** |  |
| **positive** | 21 | 0 | 21 |
| **negative** | 22 | 394 | 416 |
| **total** | 43 | 394 | 437 |

^a^Positive (gold-standard). All 43 samples were *E. coli* infection samples.

^b^Negative (gold-standard). Among the 394 samples, 272 were sterile, and the remainder were non‑*E. coli* infection samples.

**Supplementary Table 7 Comparison of detection results between T7::*Nluc***

**luminescence assay and gold-standard methods.**

| **Luminescence assay** | **Urine culture and MALDI‑TOF MS (gold-standard)** | | **total** |
| --- | --- | --- | --- |
|  | **positive^a^** | **negative^b^** |  |
| **positive** | 32 | 0 | 32 |
| **negative** | 48 | 567 | 615 |
| **total** | 80 | 567 | 647 |

^a^Positive (gold-standard). Among the 80 samples, 2 were mixed infections with *E. coli*.

^b^Negative (gold-standard). Among the 567 samples, 397 were sterile, and the remainder were non‑*E. coli* infection samples.
